# Do Strength and Tendon Stiffness Adapt in Parallel? A Systematic Review and Multilevel Meta-Analysis of Chronic Mechanical Loading in Healthy Humans

**DOI:** 10.3390/jfmk11040393

**Published:** 2026-09-29

**Authors:** Keren Pang, Zirui Li

**Affiliations:** Department of Sports & Health Science, Shinhan University, 95 Hoam-ro, Uijeongbu-si 11644, Gyeonggi-do, Republic of Korea; 20248588@o.shinhan.ac.kr

**Keywords:** tendon stiffness, muscle strength, tendon force, mechanical loading, resistance training, muscle–tendon unit, log response ratio, multilevel meta-analysis

## Abstract

**Background and Objectives:** This review evaluated whether strength and tendon stiffness show similar proportional responses to chronic mechanical loading. **Methods:** Six databases and citation chasing identified 57 reports from 50 cohorts. The primary analysis paired within-group longitudinal log response ratios (lnRRs) across 70 matched units, 62 participant samples, and 37 analytic cohorts, using multilevel models and cohort-clustered inference. This estimand was not a control-adjusted treatment effect. **Results:** Strength increased by 16.12% (lnRR 0.1495, 95% CI 0.1210 to 0.1779) and stiffness by 24.06% (lnRR 0.2156, 95% CI 0.1638 to 0.2674). The strength–stiffness contrast was −0.0661 (95% CI −0.1190 to −0.0132). Restriction to controlled designs attenuated the contrast, and its interval included zero. A reviewer-requested control-adjusted sensitivity analysis was directionally supportive but exploratory, with only 11 cohorts and low effective degrees of freedom. Strict maximal tendon strain showed no clear change (−4.77%, 95% CI −11.45% to 2.42%). **Conclusions:** The paired within-group analysis identified a small average proportional contrast, with a larger average stiffness response. The functional significance of this difference remains uncertain, and causal interpretation is limited.

## 1. Introduction

The function of the muscle–tendon unit depends on coordination between force-generating and force-transmitting tissues. Strength reflects active force-generating capacity, whereas tendon stiffness influences force transmission, fascicle operating length, elastic energy storage, and movement economy. Previous systematic reviews and meta-analyses have shown that chronic mechanical loading alters the mechanical, material, and morphological properties of healthy lower-limb tendons; effect estimates vary with loading intensity, duration, training mode, and measurement method [1,2,3].

Tendon adaptation has a mechanobiological basis. Mechanical loading can alter extracellular-matrix signaling, collagen synthesis, and remodeling, with consequent changes in tendon structure and mechanical behavior [4,5,6,7]. Age, collagen cross-linking, and tissue turnover may constrain or modify this response [8,9]. Tendon stiffness is not a directly observed, invariant material constant: it is usually derived from the tendon force–elongation relation, and estimates depend on tendon-force calculation, moment-arm correction, tendon elongation tracking, and the fitted force range [10,11,12]. Age-related differences in strength and tendon mechanics also suggest that the two outcomes may adapt on different timescales [8,13].

Neural, architectural and hypertrophic responses to strength training are well described [14,15,16,17,18], yet strength and tendon stiffness have usually been synthesized separately. Comparing separate study sets confounds outcome-specific adaptation with differences in participants and measurement. Pairing outcomes within the same cohort, participant sample, intervention arm, tendon site, and timepoint addresses this limitation. Relative changes may influence force transmission, the tendon strain environment, fascicle operating behavior, and muscle–tendon interaction [10,11,12]. However, equal proportional adaptation is not an established physiological optimum. A statistical difference in proportional adaptation is not equivalent to a physiologically meaningful muscle–tendon imbalance. No validated clinical or functional threshold currently defines a meaningful strength–stiffness adaptation gap.

This systematic review and meta-analysis quantified the paired proportional difference between strength and target-tendon stiffness after chronic mechanical-loading training in healthy humans. Secondary objectives were to evaluate strict maximal tendon strain under maximal loading, examine robustness to correlation assumptions, risk of bias, study design, and individual cohorts, and assess whether tendon site, training mode, or stiffness-fitting method explained heterogeneity.

## 2. Materials and Methods

### 2.1. Study Design and Reporting

This systematic review and multilevel meta-analysis followed PRISMA 2020 [19,20] and PRISMA-S [21]. This review was not registered. The review and statistical analyses were completed without prior public protocol registration. Internal staged extraction and analysis specifications were retained, but these do not constitute prospective registration or an authenticated prospective protocol. The original internal specifications are supplied in the reproducibility package, with their scope and subsequent changes described in Supplementary Statistical Methods. File modification times were not treated as proof of prospective specification. The reviewer-requested control-adjusted analysis is identified separately from the original primary estimand.

### 2.2. Eligibility Criteria

Eligible populations were healthy or asymptomatic adolescents and adults, including trained and untrained participants. Interventions involved repeated mechanical loading for at least 4 weeks and could include dynamic resistance, isometric, eccentric, concentric, plyometric, jumping, or mixed sport training. Parallel controlled, contralateral-limb or within-person controlled, and uncontrolled single-group pre–post designs were eligible. Mixed sport programs were eligible when they involved repeated lower-limb loading and reported the required paired outcomes; they were not assumed to deliver a uniform tendon-loading dose. The resistance-plus-stretching arm was retained as a combined intervention alongside its resistance-only comparison. Its inclusion does not identify an isolated effect of static stretching.

The core inclusion criterion was that the same participant sample reported pre–post measures of both an anatomically matched strength outcome and target-tendon stiffness for the same intervention and timepoint. For the Achilles tendon, preferred matched strength measures were Achilles tendon force, maximal voluntary plantar-flexion torque, or plantar-flexor torque. For the patellar tendon, preferred measures were patellar or quadriceps tendon force, quadriceps strength, maximal voluntary knee-extension torque, or knee-extensor torque. Jump height, sprint performance, and anatomically unrelated one-repetition maximum tests were not accepted as matched strength outcomes on their own. Tendon strain was classified as strict maximal strain only when assessed during maximal voluntary contraction, maximal tendon force, maximal loading, or 100% maximal torque.

We excluded animal studies, acute-loading studies, pathological or postoperative rehabilitation populations, immobilization or unloading studies, cross-sectional studies, studies without target-tendon stiffness, studies without anatomically matched strength, studies without longitudinal pre–post data, and studies in which the two outcomes could not be paired within the same sample.

### 2.3. Information Sources and Search Strategy

PubMed/MEDLINE was searched through NCBI PubMed E-utilities up to 13 August 2026. Web of Science Core Collection and SPORTDiscus, CINAHL Ultimate, Rehabilitation & Sports Medicine Source and Academic Search Premier (EBSCOhost) were searched through 14 August 2026. Searches covered database inception to the recorded search date; the PubMed query explicitly used a publication-date interval beginning in 1800. No language filter was applied, and strength terms were not mandatory. Concepts covered tendon anatomy and mechanics, repeated training/loading, and longitudinal adaptation. The supplementary EBSCO databases used a focused anatomical/mechanics query. Table S4 reproduces the retained search records, including field tags, dates, filters, and counts. For CINAHL Ultimate, Rehabilitation & Sports Medicine Source and Academic Search Premier, complete contemporaneous search instructions were recovered from the retained project record and are reproduced as separate TI/AB blocks with N2 proximity operators. Database-tagged exports corroborate the recorded totals.

The six database searches identified 25,699 raw records in total: 7586 in PubMed, 13,945 in Web of Science, 3323 in SPORTDiscus, 137 in CINAHL Ultimate, 184 in Rehabilitation & Sports Medicine Source, and 524 in Academic Search Premier. Search results were processed sequentially rather than pooled in a single batch. PubMed formed the initial screening pathway; subsequent exports were deduplicated against the existing master database, and only genuinely new records were screened. The five additional databases contributed 18,113 raw records, of which 8837 were exact matches to the existing master, and 9276 were genuinely new records; 8674 of the new records were excluded at title and abstract screening, and 602 entered a further eligibility-adjudication queue. Database attribution used source tags so that SPORTDiscus records contained in a mixed EBSCO export were not counted twice. Scopus was not searched because access was unavailable; the reproducible search strategy was retained, and this omission was considered when interpreting search completeness. We performed backward reference checking and Web of Science forward citation chasing using included reports and key systematic reviews as seeds. The final Web of Science backward export matched 55 seeds; complete citation-index coverage of every intended seed was not documented. One further PubMed-indexed backward and forward pass for the newly included Quinlan report identified no additional eligible cohort. Citation-chasing closure was recorded on 15 August 2026.

### 2.4. Study Selection and Report Retrieval

Records were deduplicated in descending priority using DOI, PMID, exact normalized title, and title plus first author and year. Fuzzy matches were flagged as possible duplicates and resolved manually. Title and abstract screening used a high-recall approach. Absence of a strength outcome, detailed stiffness method, or complete numerical data from the abstract was not an automatic reason for exclusion. Retained records underwent report-level eligibility assessment.

Full reports were sought through PubMed Central, publishers, institutional repositories, author manuscripts, and Supplementary Files. Potentially eligible reports that could not be obtained were classified as reports not retrieved, not exclusions. Zirui Li conducted title/abstract screening, and Keren Pang conducted full-text eligibility assessment. The researchers made initial judgments within their assigned stages; this was not independent duplicate screening of every record at both stages. Disputed judgments were discussed, and unresolved disagreements were adjudicated by Yixin Cui. AI-assisted preliminary classification supported a high-recall workflow, but the absence of strength or detailed mechanics in an abstract was not sufficient for exclusion. Human reassessment of AI-assisted exclusions focused on disputed records; exhaustive duplicate checking of every AI classification was not documented. AI did not independently make final eligibility decisions.

### 2.5. Biological Identity and Duplicate-Cohort Handling

Before analysis, we mapped each publication to its cohort, participant sample, and effect arm. Author lists, sample size, age, sex, training protocol, baseline characteristics, tendon site, and follow-up duration were compared to identify companion publications and repeated cohorts. Companion reports could supply additional data or methodological detail for the same biological effect but did not create an additional independent effect.

The biological analysis key comprised cohort, participant sample, effect arm, tendon site, outcome, and timepoint. Report identifiers were retained solely for provenance. Parallel groups containing different participants could contribute separate participant samples. Within-person or contralateral-limb arms from the same participants retained separate effect estimates but were treated as dependent.

### 2.6. Data Extraction and Numerical Recovery

We extracted sample size, pre-training and post-training means and standard deviations, reported change scores and their dispersion, and standard errors, confidence intervals, *p*-values, or test statistics that could support permitted recovery. Values reported directly in text or tables were preferred. Standard errors of the mean were converted to standard deviations with provenance retained. Formal figure digitization was performed only when the figure reliably supported recovery of the required mean and dispersion, with page, figure, and source recorded.

A missing post-training standard deviation was not replaced with a change-score standard deviation, and no equal-standard-deviation assumption was introduced. Pre–post correlations were recorded when reported or derived from a reported change-score standard deviation. No correlation was imputed during extraction.

We recorded the strength endpoint, tendon-force estimation method, moment-arm correction, tendon elongation method, stiffness-fitting range, strain definition, training duration, and intervention type. The frozen strength priority was maximal target-tendon force derived from the matched MVC test, followed by the matched joint MVC/isometric peak torque used for tendon-mechanics testing, and then another anatomically matched maximal endpoint if neither was available. Alternate endpoints were not averaged. Stiffness selection prioritized the report’s prespecified target-tendon force–elongation slope under active loading; when both common absolute and timepoint-relative ranges were available, the common high-force range was preferred, followed otherwise by the report’s primary high-force linear-region estimate. Young’s modulus was not substituted for stiffness. Bilateral intervention effects retained their common participant-sample identifier, and the first formal endpoint after training completion was primary; longer follow-up was excluded from the primary contrast. The complete rules and recorded method fields are supplied in Table S5 and Supplementary Statistical Methods and the accompanying extraction files.

### 2.7. Risk-of-Bias Assessment

Risk-of-bias tools were assigned according to the actual study design. Randomized parallel controlled and randomized within-person studies were assessed with the RoB 2 framework [22], non-randomized controlled interventions with ROBINS-I [23], and uncontrolled single-group pre–post studies with the National Heart, Lung, and Blood Institute (NHLBI) Quality Assessment Tool for Before-After (Pre-Post) Studies With No Control Group [24]. For randomized within-person or contralateral-limb comparisons, randomization referred to limb-level allocation; the standard RoB 2 domains were applied to that assigned comparison, and limb dependence and potential cross-limb contamination were recorded in the assessment notes. Non-randomized within-person studies were assessed with ROBINS-I. Moment-arm methods, ultrasound techniques, tendon-force estimation, and stiffness-fitting ranges were treated primarily as sources of measurement heterogeneity and were coded as bias only when they caused differential measurement, systematic assessor bias, or inconsistent pre–post measurement. Tool-specific domain and overall judgments were retained without conversion to a cross-tool numerical quality score. Keren Pang and Zirui Li independently assessed risk of bias. Disagreements were resolved through discussion or, when consensus could not be reached, adjudication by Yixin Cui. Companion reports supplemented a single biological study assessment.

### 2.8. Effect Sizes and Primary Statistical Model

For each outcome, m_0_ and m_1_ denote pre-training and post-training means, s_0_ and s_1_ their standard deviations, and n the outcome-specific analyzed sample size. The longitudinal log response ratio and first-order sampling variance were calculated as follows [25,26]. Reported pre–post correlations were preferred; when available, correlations were derived from change-score dispersion. Otherwise, r = 0.70 was used, with sensitivity values of 0.30, 0.50 and 0.90. Means and effect sizes were unchanged across these variance scenarios.

yᵢ = ln(m_1_/m_0_); vᵢ = s_0_^2^/(n m_0_^2^) + s_1_^2^/(n m_1_^2^) − 2r s_0_s_1_/(n m_0_m_1_).

r = (s_0_^2^ + s_1_^2^ − sΔ^2^)/(2s_0_s_1_); SD = SEM × √n.

One strength lnRR and one stiffness lnRR were paired within the same cohort, participant sample, effect arm, tendon site, and timepoint. The primary estimand was the adaptation gap, delta = mu_strength − mu_stiffness. Positive values indicated greater proportional strength adaptation, negative values indicated greater proportional stiffness adaptation, and zero indicated no average difference between the two within-group lnRRs. The first formal post-training timepoint was used for the primary analysis; longer-term follow-up data were retained as non-primary longitudinal information. This contrast describes within-group longitudinal changes and is not a control-adjusted estimate of the training effect.

Models were fitted in R using metafor and restricted maximum likelihood [27]. For outcome o in matched unit u, participant sample s and cohort c, the model was y_csu,o = μ_o + b_c + a_cs + h_csu,o + e_csu,o. The cohort random intercept b_c captured shared cohort-level variation, the sample random intercept a_cs captured variation shared by units from the same participants, and h_csu,o allowed outcome-specific heterogeneity within each matched unit. These random components were independent, with a diagonal covariance structure for h. Fixed effects were specified as outcome indicators without an intercept. Sampling errors e followed a block-diagonal working covariance matrix: each matched unit contributed a two-outcome block with diagonal entries v_strength and v_stiffness and off-diagonal covariance ρ√(v_strength v_stiffness). The primary ρ was 0.50; sensitivities used 0, 0.30, 0.50, 0.70, and 0.90. Unknown sampling covariances between different units were set to zero in this working matrix. Participant/cohort random effects and cohort-clustered inference accommodated dependence but did not identify the unreported sampling covariances.

Primary inference used cohort-clustered CR2 robust variance estimation with Satterthwaite degrees of freedom [28,29,30]. Cohorts, rather than matched units, defined the independent inference clusters. Heterogeneity is reported as variance components and their square-root standard deviations on the lnRR scale [31,32,33]. A reporting-only, model-conditional prediction interval for the true contrast in a new matched unit was obtained from the frozen output as δ ± t(0.975, ν)√[SE_CR2(δ)^2^ + τ_strength^2^ + τ_stiffness^2^]. Shared cohort and sample intercepts cancel in the within-unit contrast, and the two DIAG outcome-specific variances add. This plug-in approximation does not propagate uncertainty in the estimated heterogeneity components; it is not a population-wide clinical threshold.

### 2.9. Sensitivity Analyses, Moderators, and Small-Study Effects

Core sensitivity analyses varied the cross-outcome and pre–post correlations, excluded RoB 2 high-risk and ROBINS-I serious-risk cohorts, retained controlled designs only, and omitted one cohort at a time. The controlled-design sensitivity restricted the evidence base to controlled study designs but retained the within-group longitudinal estimand; it was not a control-adjusted treatment-effect analysis. A targeted mechanical-loading-dominant sensitivity analysis excluded active nutritional or pharmacological adjunct arms involving acetaminophen, ibuprofen, collagen, or specific collagen peptides. Placebo arms and exposure strata not introduced as tendon-targeted adjuncts by the trial were retained. Moderator variables specified before model fitting were tendon site, training or loading mode, and stiffness-fitting method. A moderator test was performed only when a category contained at least eight matched units and five cohorts, and false-discovery-rate correction was applied across formal tests. Multi-parameter interaction tests used the approximate Hotelling’s T^2^-Z (HTZ) small-sample Wald test [28,29,30]. Reference categories were patellar tendon, dynamic resistance training, and individualized relative/maximal fitting range. Category-specific unit, sample, and cohort counts are provided in the Supplementary Materials; sparse categories were not interpreted as equivalent to better-represented categories.

In response to Reviewer 2, an available-data control-adjusted sensitivity analysis used outcome-specific contrasts L_training − L_control, where L is the longitudinal lnRR. Concurrent comparators were not assumed to be inactive. The sampling covariance matrix was constructed as V = BΣBᵀ: Σ contains the variances and working cross-outcome covariances of the unique training and control estimates, and B encodes +1 training and −1 control contributions. Reusing the same control induces covariance algebraically rather than duplicating its sample size. The primary settings were r = 0.70 and ρ = 0.50, with the frozen cross-outcome correlation grid 0, 0.30, 0.50, 0.70 and 0.90. Reported/derived pre–post correlations took priority. This exploratory analysis retained the frozen multilevel/REML and cohort-CR2 procedures; its results are distinct from the controlled-design-restricted within-group analysis.

We did not treat the 70 matched units as independent studies in an ordinary Egger test. For each unit, the adaptation gap was g = y_strength − y_stiffness, with working variance v_g = v_strength + v_stiffness − 2ρ√(v_strength v_stiffness). We regressed g on √v_g in a multilevel model retaining cohort and participant-sample dependence, with cohort-clustered CR2 inference. The diagnostic involved 37 independent cohort clusters. Its power is limited by the number of independent clusters, heterogeneity, and assumed sampling correlations; a non-significant result cannot rule out small-study effects or publication bias [34]. Risk-of-bias visualization retained tool-specific judgments [35]. Methodological decisions were also informed by the Cochrane Handbook [36].

## 3. Results

### 3.1. Study Selection and Characteristics

**Figure 1:**
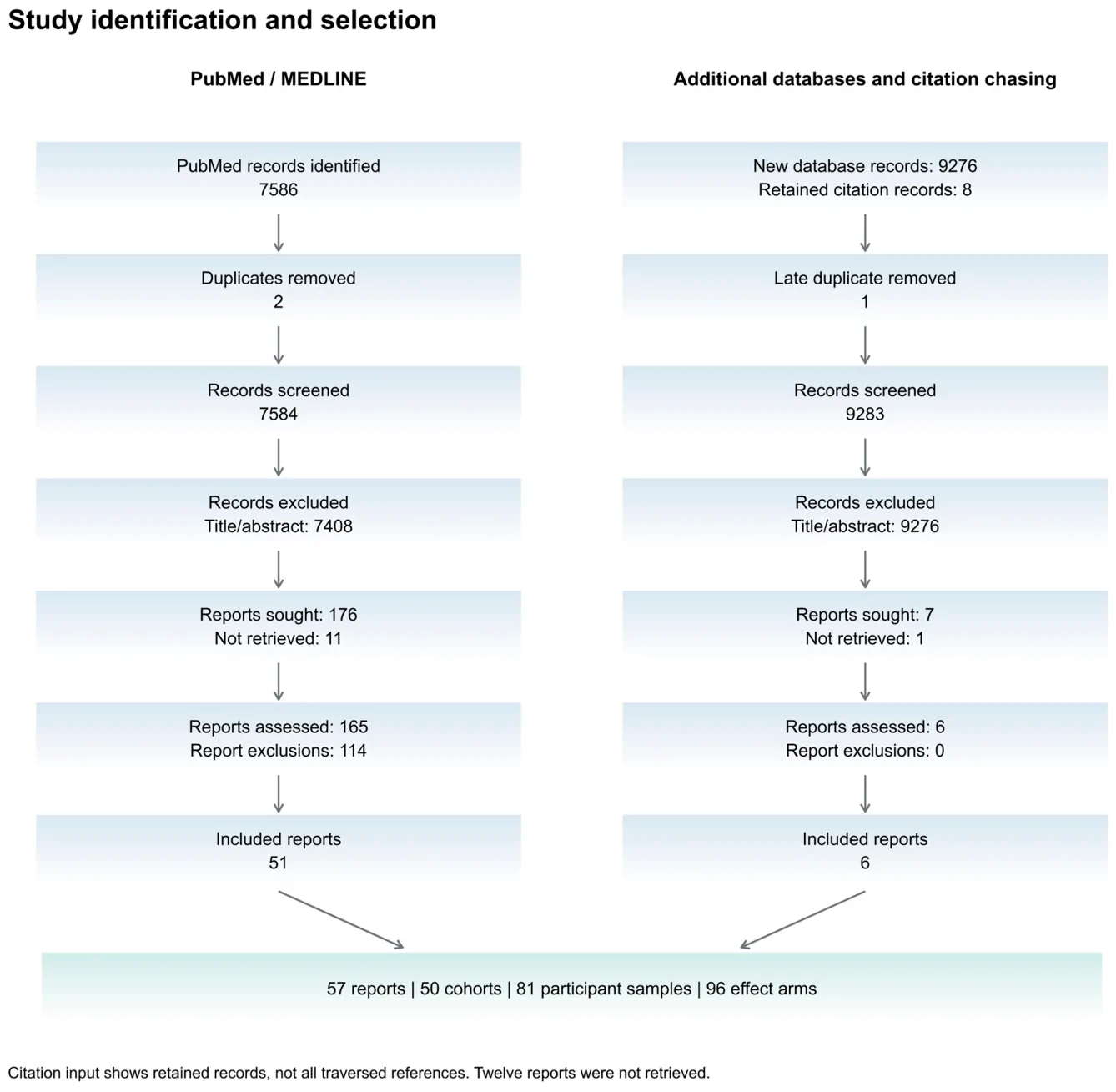
Study identification and selection using final decision-stage terminology. PubMed and subsequent database/citation pathways are shown separately. The citation input on the right comprises eight retained records, not all references traversed. The late duplicate is assigned before the final effective screening counts. Decisive title/abstract exclusions are not counted as full-text exclusions. The pathways yield 57 included reports, 50 cohorts, 81 participant samples and 96 effect arms. Twelve reports were not retrieved and contributed to none of these included identities. Scopus was not searched because access was unavailable. Detailed source and operational counts are provided in Table S4.

Across the six databases, 25,699 raw records were identified before cross-database deduplication. Within the initial PubMed pathway, removal of two internal duplicates left 7584 records for screening. Of these, 7408 were excluded at title and abstract level, and 176 reports were sought for retrieval. Eleven reports were not retrieved, 165 were assessed for eligibility, 114 were excluded after report-level assessment, and 51 were included. The five additional databases contributed 18,113 raw records; sequential deduplication identified 8837 exact matches to the existing master, leaving 9276 genuinely new records for screening. Of these, 8674 were excluded at title and abstract level, and 602 entered a further eligibility-adjudication queue. Seven records retained through citation chasing were combined with these 602 records, yielding a 609-record adjudication queue. This queue produced three new primary reports, two informative companion reports, 602 exclusions based on decisive title/abstract information, one report not retrieved, and one database duplicate that was merged. Bounded backward and forward citation chasing subsequently identified one further included report. The final set comprised 57 reports [37,38,39,40,41,42,43,44,45,46,47,48,49,50,51,52,53,54,55,56,57,58,59,60,61,62,63,64,65,66,67,68,69,70,71,72,73,74,75,76,77,78,79,80,81,82,83,84,85,86,87,88,89,90,91,92], representing 50 cohorts, 81 independent participant samples, and 96 effect arms (Figure 1). Twelve potentially relevant reports were not retrieved, including 11 from the PubMed phase and one from the Web of Science phase. None contributed to the included-report, cohort, sample, or arm counts. These 602 exclusions were classified by their decision basis and must not be counted as reports excluded following full-text assessment. The 609-record workflow count is therefore not a count of full texts assessed.

The corrected identity ledger contained 34 Achilles and 52 patellar participant-sample records; site counts overlap and are not additive. No eligible quadriceps tendon sample was identified. The 50 cohorts comprised 20 randomized parallel, 5 randomized within-person, 12 non-randomized parallel, 5 non-randomized within-person, and 8 uncontrolled single-group pre–post designs. Albracht 2013 [43] and Bohm 2021 [79] are represented independently. The review retained repeated chronic mechanical-loading interventions rather than assuming a uniform training dose.

Of 81 review-level participant samples, 62 contributed fully variance-calculable paired outcomes to the final primary set. The remaining 19 did not contribute to this set because of insufficient numerical information or the frozen endpoint-selection rules. The latter includes removal of an anatomically ineligible composite strength endpoint during source verification. Thus, non-contribution to the final analytic set is not synonymous with numerical extraction failure. Missing post-training dispersion was not replaced with change-score dispersion or guessed values.

Table 1 summarizes the 37 analytic cohorts. Table S1 provides separate participant, limb, arm, and matched-unit fields, existing demographic/loading characteristics, and available technical measurement information. NR denotes explicitly not reported; NE denotes not extracted or unavailable in the retained extraction record. NE is not evidence that the original publication omitted the information. Targeted source verification recovered previously unextracted Table S1 characteristics without changing eligibility, biological identity, risk-of-bias judgments, primary effect sizes, or frozen sensitivity analyses. Only the previously documented training-mode moderator correction arose from this verification.

### 3.2. Risk of Bias

Risk-of-bias judgments were completed for 44 of 50 cohorts; 6 remained unresolved owing to insufficient source information (Figure S1). RoB 2 was allocated to 25 cohorts: three low risk, 20 some concerns, one high risk, and one unresolved. ROBINS-I was allocated to 17 cohorts: five moderate, seven serious, and five unresolved. All eight uncontrolled cohorts received NHLBI before–after judgments: three good and five fair. These totals were generated from the corrected ledger, including author-confirmed Bohm 2021 [79] (RoB 2, some concerns) and Albracht 2013 [43] (ROBINS-I, serious). The six unresolved cohorts did not contribute to the primary analysis. The scarcity of low-risk randomized evidence and serious-risk non-randomized evidence limits confidence independently of the sensitivity *p*-values.

### 3.3. Primary Strength–Stiffness Adaptation

**Figure 2:**
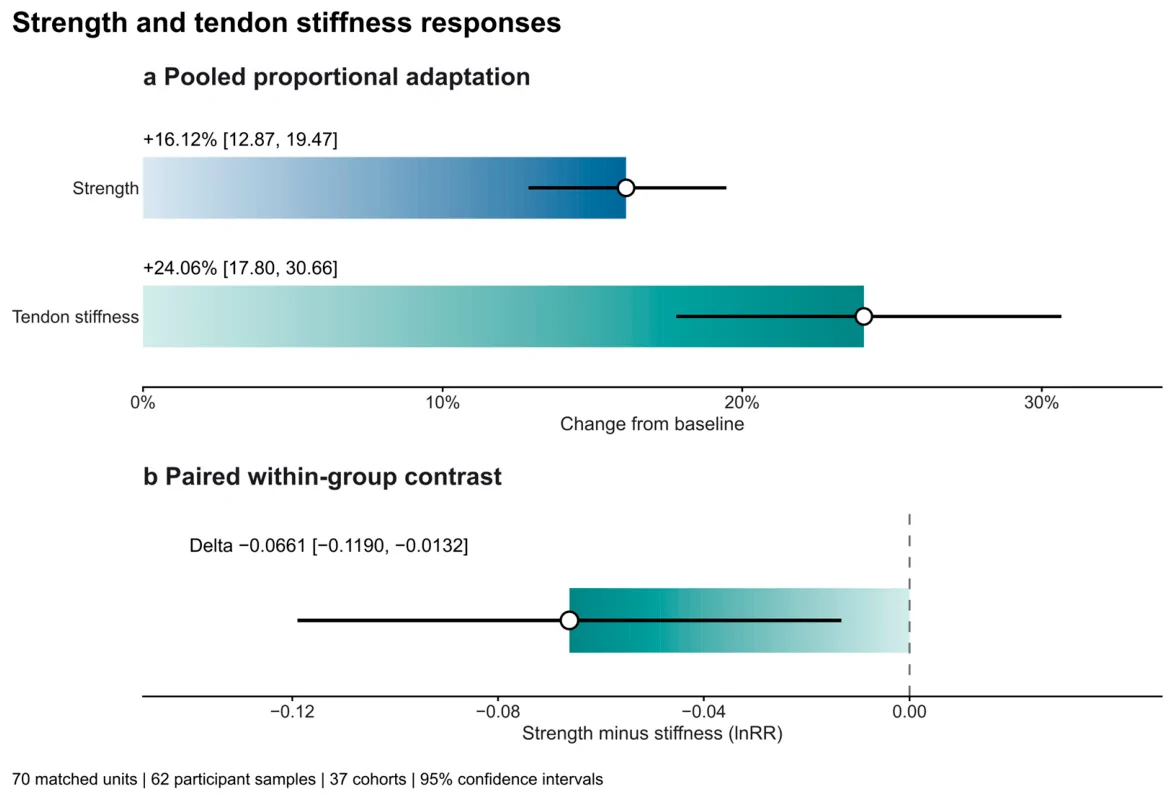
Primary paired adaptations in strength and tendon stiffness. (**a**) Pooled natural-scale changes in strength and tendon stiffness with 95% confidence intervals. (**b**) The adaptation gap, delta, calculated as strength minus stiffness; negative values indicate a greater proportional stiffness adaptation. The primary analysis used the first formal post-training timepoint, r = 0.70, rho = 0.50, and cohort-clustered CR2 inference.

The pooled strength lnRR was 0.1495 (95% CI 0.1210 to 0.1779), corresponding to +16.12% (95% CI +12.87% to +19.47%). The pooled tendon-stiffness lnRR was 0.2156 (95% CI 0.1638 to 0.2674), corresponding to +24.06% (95% CI +17.80% to +30.66%; Figure 2).

**Figure 3:**
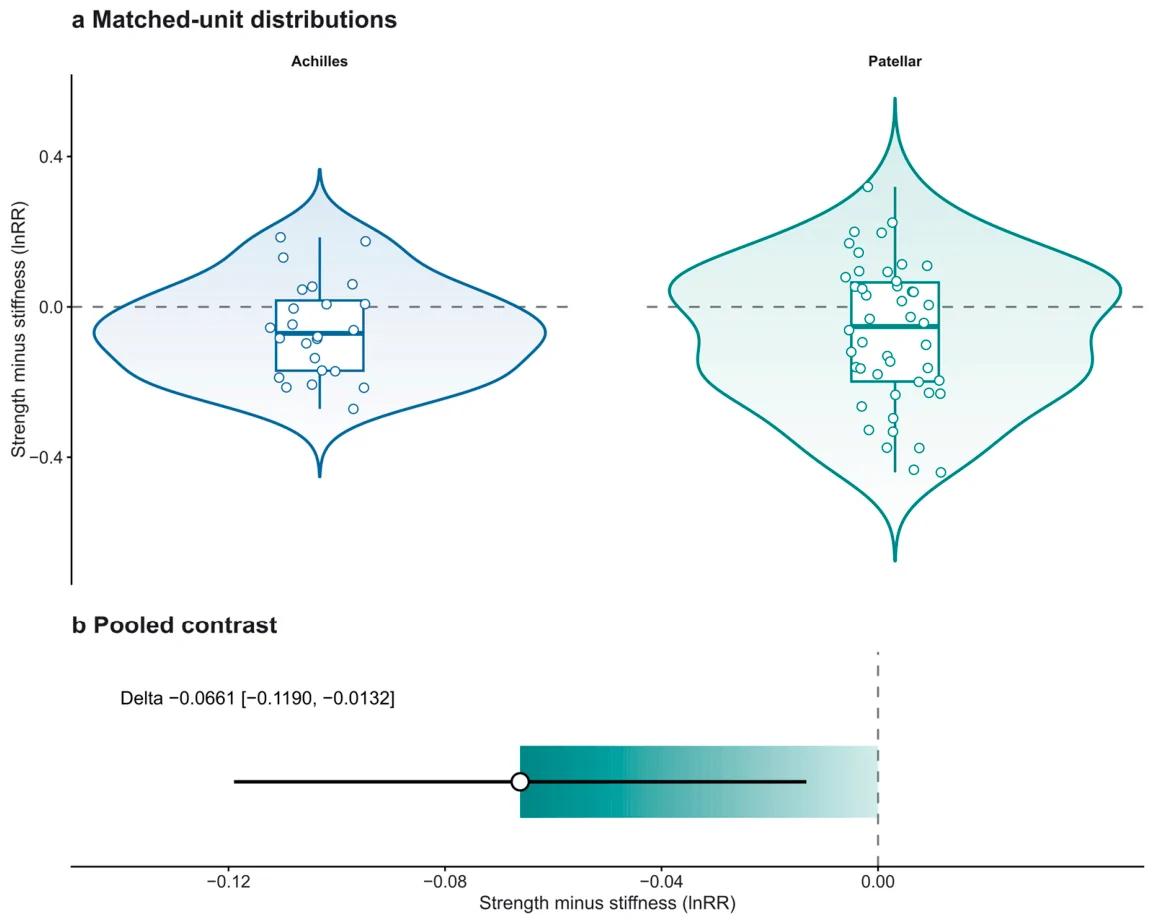
Distribution of the matched adaptation gap by tendon site. (**a**) Points represent matched biological units, and shaded distributions summarize the adaptation gap for Achilles and patellar tendons. (**b**) The primary pooled delta and its 95% confidence interval are shown separately. Units are correlated within participant samples and cohorts.

The primary adaptation gap was −0.0661 lnRR units (95% CI −0.1190 to −0.0132; CR2 SE 0.02592; Satterthwaite df 30.81; *p* = 0.0159). The ratio of response ratios, exp(δ), was 0.9360: the proportional strength response was approximately 6.4% lower than the proportional stiffness response. This is not a 6.6-percentage-point difference, a functional threshold, or evidence of causal biological uncoupling. Figure 3 displays the final 70 matched units by tendon site.

### 3.4. Heterogeneity and Robustness

The frozen primary model estimated cohort-level heterogeneity SD 0.0640 (variance 0.004101), participant-sample SD approximately zero, strength-specific SD 0.0451 (variance 0.002032) and stiffness-specific SD 0.1424 (variance 0.020273). Equality of components was not tested. The model-conditional 95% prediction interval for a true contrast in a new matched unit was −0.375 to 0.243. It permits either direction and depends on the random-effects structure and plug-in heterogeneity estimates.

**Figure 4:**
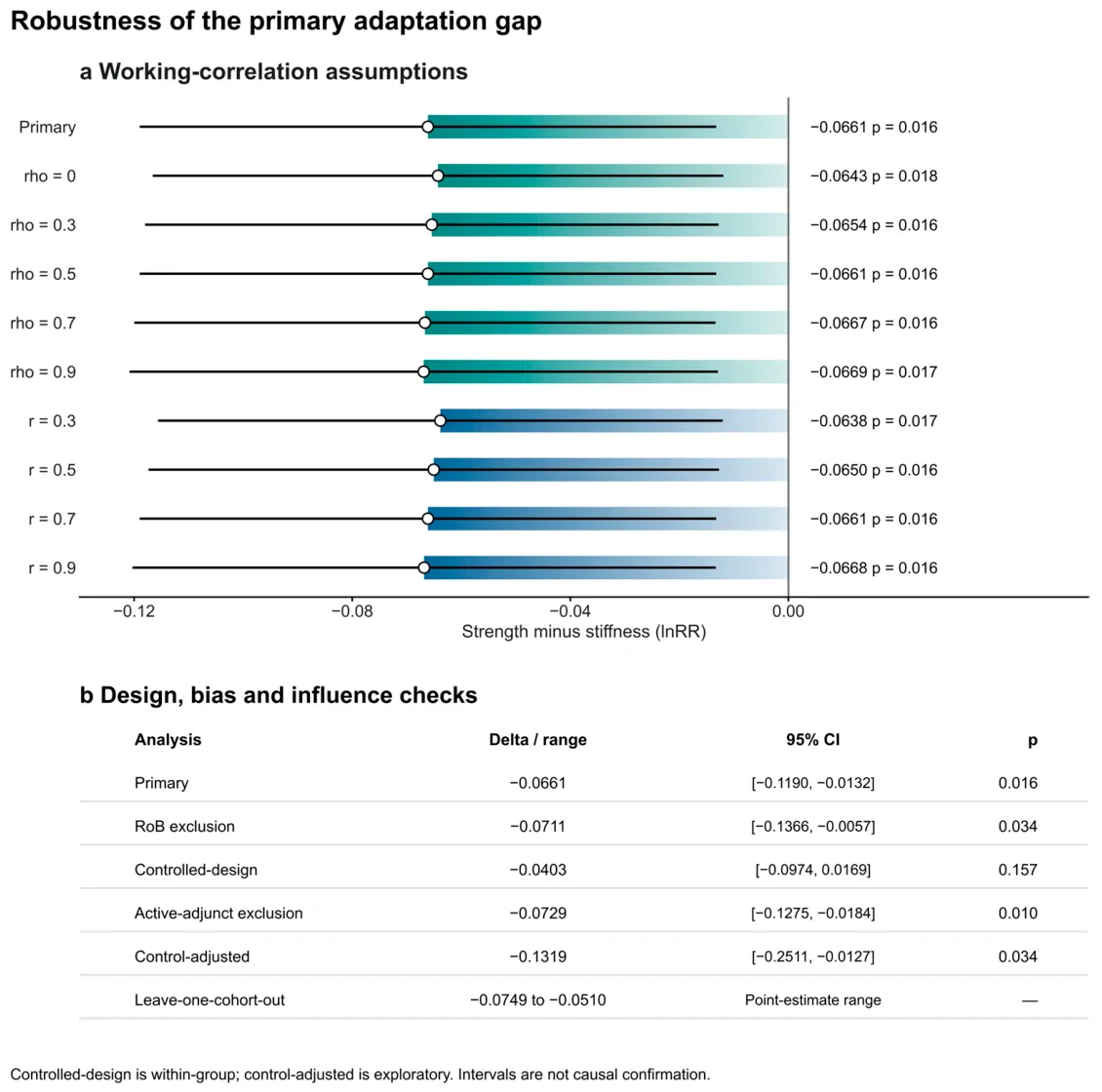
Robustness of the primary adaptation gap. (**a**) Frozen primary and working-correlation scenarios with 95% confidence intervals. (**b**) Frozen RoB, controlled-design, active-adjunct, control-adjusted, and leave-one-cohort-out results. Ranges denote point-estimate ranges, not pooled confidence intervals. Controlled-design restriction retains a within-group estimand; control adjustment estimates a different contrast. All numbers use corrected final outputs.

Across ρ = 0 to 0.90, delta ranged from −0.0669 to −0.0643; across pre–post r = 0.30 to 0.90, it ranged from −0.0668 to −0.0638. All estimates remained negative and all corresponding confidence intervals excluded zero (Figure 4). RoB exclusion retained 56 units from 50 samples and 30 cohorts: δ = −0.0711 (95% CI −0.1366 to −0.0057; df 25.42; *p* = 0.034).

The controlled-design-restricted analysis retained 47 units from 42 participant samples and 23 cohorts. It restricted study design while retaining within-group longitudinal changes; it was not a control-adjusted treatment-effect analysis. The contrast was δ = −0.0403 (95% CI −0.0974 to 0.0169; df 19.56; *p* = 0.157). The attenuated estimate and interval including zero make the average contrast less conclusive in this subset.

The reviewer-requested available-data control-adjusted sensitivity included 18 intervention units, 18 intervention samples, 11 cohorts, and 11 concurrent control groups. It yielded δ = −0.1319 (95% CI −0.2511 to −0.0127; df 8.62; *p* = 0.0338; exp(δ) = 0.8764). This point estimate was directionally supportive, not confirmatory. Only 11 independent cohorts, effective df below 10, heterogeneous comparators, and a confidence interval crossing zero at ρ = 0.90 limit causal interpretation (Table S3).

Excluding eight active nutritional or pharmacological adjunct units retained 62 units from 55 samples and 37 cohorts. The contrast was δ = −0.0729 (95% CI −0.1275 to −0.0184; df 30.57; *p* = 0.010). Placebo arms were retained. This analysis did not eliminate all possible co-intervention effects.

All 37 frozen leave-one-cohort-out models converged. Delta ranged from −0.0749 to −0.0510; there were 0 direction reversals and 0 intervals including zero. The maximum absolute deviation was 0.0151. The small-study diagnostic used 70 units clustered within 37 independent cohorts and yielded a working-SE slope of −1.391 (95% CI −4.338 to 1.557; df 14.75; *p* = 0.330; Figure S2). There was no clear evidence of small-study effects. The modest cluster count, limited effective degrees of freedom, and precision–heterogeneity association limit power; this result does not establish the absence of publication bias.

### 3.5. Moderator Analyses

The final primary site categories comprised 24 Achilles units from 15 cohorts and 46 patellar units from 23 cohorts; overlapping cohorts make counts non-additive. With patellar tendon as reference, the Achilles interaction was 0.0071 (95% CI −0.0951 to 0.1092; *p* = 0.888). The formal training comparison retained dynamic resistance (40 units/22 cohorts, reference), eccentric-dominant loading (8/6), isometric loading (10/8), and plyometric/sport-mixed loading (10/6); other/mixed (2/2) remained descriptive. The approximate Hotelling’s T^2^-Z test yielded F = 0.683, df = 3 and 10.26, *p* = 0.582, FDR *p* = 0.873. The fitting comparison retained common absolute ranges (16/9) and individualized relative/maximal ranges (33/17, reference); study-reported/unclear units (21/11) were excluded from that formal contrast. The interaction was 0.0886 (95% CI −0.0534 to 0.2307; *p* = 0.202; FDR *p* = 0.607; Table S2). These results indicate limited evidence of moderation, not equivalence.

### 3.6. Strict Maximal Tendon Strain

**Figure 5:**
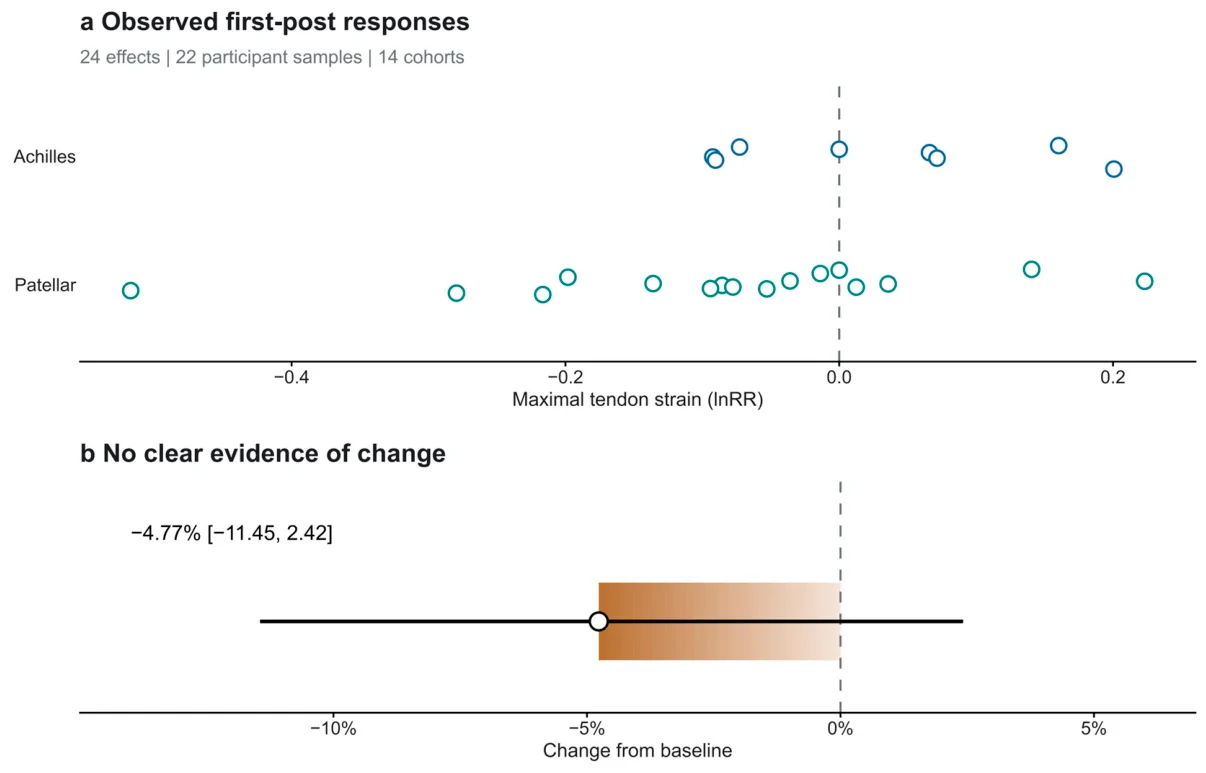
Strict maximal tendon strain as a key secondary outcome. (**a**) First-post observations for 24 effects from 22 participant samples and 14 cohorts. (**b**) Pooled natural-scale change and 95% confidence interval, transformed from the frozen lnRR output. The interval includes zero: there was no clear evidence of a change.

The first-post strict maximal-strain analysis included 24 effects from 22 participant samples and 14 cohorts. Pooled lnRR was −0.0489 (95% CI −0.1216 to 0.0239; CR2 SE 0.03349; df 12.36; *p* = 0.170), corresponding to −4.77% (95% CI −11.45% to +2.42%; Figure 5). There was no clear evidence of a change in strict maximal tendon strain. The definition requires maximal contraction or loading, not merely a common absolute force. Sharing a maximal-force test or force signal does not prove that strain and the chosen strength endpoint came from the same contraction trial; same-trial identity was not consistently documented in the retained extraction records. Source definitions and observed measurement coupling are retained in Table S5 and the source-mapping files.

Core pooled and sensitivity estimates are summarized in Table 2.

## 4. Discussion

### 4.1. Principal Findings

Strength and tendon stiffness both increased. Their paired within-group contrast was small (δ = −0.0661; ratio of response ratios 0.9360), with a larger average stiffness response. This does not identify a percentage-point difference or a functional threshold. The controlled-design estimate was attenuated, and its interval included zero, deserving substantial weight in interpretation. The available-data control-adjusted point estimate was directionally supportive but exploratory: 11 cohorts, low effective degrees of freedom, heterogeneous comparators, and sensitivity to a high cross-outcome correlation prevent a confirmatory causal claim. Statistical evidence for a proportional difference does not establish a physiologically harmful muscle–tendon imbalance.

### 4.2. Biological and Training Interpretation

Tendon remodeling depends on loading magnitude, duration, and rhythm [4,5,6,7]. Studies of Achilles and patellar tendons motivate examining coordinated responses [38,39,40,41,42,43,44,45,46,47,48,49,50], but this synthesis cannot distinguish biological adaptation from differences in loading dose or outcome construction. It also cannot determine which tissue adapts faster: the analysis compares responses at a post-training endpoint, not adaptation rates.

Neural, hypertrophic, and architectural responses contribute to strength [14,15,16,17,18], whereas stiffness reflects tissue geometry, material behavior, and the fitted force range [4,5,6,7,8,9,10,11,12]. Equal proportional changes are not an established physiological optimum. No validated clinical or functional threshold defines a meaningful strength–stiffness adaptation gap. Whether a particular difference affects movement economy, force transmission, or fascicle behavior requires direct functional evidence. Age, training status and prior loading may further alter interpretation [58,62,65,70,71,75,83,92].

### 4.3. Tendon Strain and Potential Functional Relevance

Strict maximal tendon strain showed no clear average change. The uncertainty allows either direction and does not support a protective-strain claim. Interpretation depends on maximal versus common-force loading, reference length and measurement procedures. Strain, force and stiffness may share a force ramp or measurement signal; their association therefore cannot be assumed to reflect independent biological coupling. Studies should report whether outcomes came from the same contraction trial.

### 4.4. Moderators and Methodological Heterogeneity

Moderator findings provided limited evidence that tendon site, training mode, or stiffness-fitting category explained the gap. Sparse categories and imprecise interactions preclude equivalence claims. Method categories do not isolate every difference in force estimation, moment-arm correction, or ultrasound tracking. These differences were treated as measurement heterogeneity rather than automatic risk of bias unless they implied differential or systematically biased assessment.

### 4.5. Methodological Strengths

Biological-unit pairing and companion-report consolidation reduced avoidable duplication. Multiple arms and tendon sites retained participant-sample dependence, and inference used cohort clusters [28,29,30]. The review combined six databases and bounded citation chasing. Extraction retained source provenance and distinguished post-training from change-score dispersion. Source verification corrected an ineligible composite endpoint and separated Albracht 2013 [43] from Bohm 2021 [79] before final analyses were frozen.

### 4.6. Limitations

The review and statistical analyses were completed without prior public protocol registration, and the review remains unregistered. This limits independent verification of prespecified methods; internal specifications cannot provide equivalent protection against selective decisions. Screening responsibilities were divided across stages, and human reassessment of AI-assisted exclusions focused on disputed records. Missed eligible reports remain possible. Scopus was unavailable, complete citation-index seed coverage was not documented, and 12 potentially relevant reports were not retrieved. Only 62 of 81 review-level participant samples contributed to the final primary set, so numerical availability and endpoint eligibility may limit representativeness.

Most studies did not report the correlations required to identify sampling covariance. Working assumptions and cluster-robust inference cannot eliminate covariance misspecification or distinguish biological dependence from shared force/torque inputs. Within-group changes remain susceptible to familiarization, secular trends, and co-interventions, even in studies with controls. The controlled-design restriction and the exploratory control-adjusted sensitivity answer different questions. Neither the latter’s nominal *p*-value nor exclusion of active-adjunct arms removes these limitations.

The evidence profile further limits confidence: only three randomized cohorts were low risk, 20 had some concerns, seven non-randomized cohorts had serious risk, and six review-level assessments remained unresolved. Exclusion sensitivity cannot remove biases shared by retained studies. Heterogeneity in measurement methods [10,11,12] and a new-unit prediction interval spanning both directions caution against assuming a common response across settings. No eligible quadriceps tendon sample was identified. The small-study diagnostic also had limited statistical power and cannot establish the absence of publication bias [34].

### 4.7. Implications for Practice and Research

The pooled contrast does not justify a target strength–stiffness ratio, a clinical decision threshold, or an injury-risk marker. Future controlled trials should assess anatomically matched strength, stiffness and strain together, report loading dose and comparator activity, distinguish maximal from common-force measurements, and identify shared contraction trials. Complete pre/post and change-score dispersion, covariance information and reusable group-level data would enable more informative causal and mechanistic comparisons.

## 5. Conclusions

Strength and tendon stiffness both increased, with a small average paired within-group proportional contrast favoring a larger stiffness response. The controlled-design estimate was attenuated and imprecise. An available-data control-adjusted sensitivity was directionally supportive but limited by its small, heterogeneous evidence base and low effective degrees of freedom. Functional and clinical significance remain uncertain; the results do not establish harmful imbalance or causal uncoupling. Strict maximal tendon strain showed no clear change.

## Figures and Tables

**Table 1:** Characteristics of cohorts contributing to the primary analysis.

Study	Tendon	Design	Loading	Weeks	Source n	Units
Seynnes 2009 [40]	Patellar	Uncontrolled single group prepost	HEAVY KNEE EXTENSION RESISTANCE TRAINING	9	15	1
Bohm 2021 [79]	Achilles	Randomized parallel controlled	Isometric plantar flexion 90% MVC plus habitual running; 3–4/week; 5 × 4; 3 s on/off; supervised	14	13	1
Reeves 2003 [37]	Patellar	Uncontrolled single group prepost	HEAVY RESISTANCE	14	9	1
Arampatzis 2007 [38]	Achilles	Randomized within-person	LOW STRAIN; HIGH STRAIN	NE	11	2
Kongsgaard 2007 [39]	Patellar	Randomized within-person	LIGHT RESISTANCE; HEAVY RESISTANCE	12	12	2
McMahon 2013 [45]	Patellar	Randomized parallel controlled	RESISTANCE TRAINING MTCS; RESISTANCE TRAINING MTCL; RESISTANCE TRAINING MTCX	8	10; 11	3
Kubo 2012 [42]	Achilles	Uncontrolled single group prepost	ISOMETRIC PLANTAR FLEXION TRAINING	12	9	1
Kubo 2017 [47]	Achilles	Randomized within-person	PLYOMETRIC; ISOMETRIC	12	11	2
Malliaras 2013 [44]	Patellar	Randomized parallel controlled	CONCENTRIC; STANDARD ECCENTRIC; HIGH LOAD ECCENTRIC	12	9; 10	3
Kubo 2017 [48]	Patellar	Randomized within-person	CONCENTRIC; ECCENTRIC	NE	7	2
Massey 2018 [50]	Patellar	Randomized parallel controlled	Explosive-contraction isometric knee-extension training (ECT); sustained-contraction isometric knee-extension training (SCT)	12	13; 15	2
Geremia 2018 [49]	Achilles	Uncontrolled single group prepost	HIGH LOAD ECCENTRIC PLANTAR FLEXION TRAINING	12	15	1
McMahon 2018 [51]	Patellar	Non-Randomized parallel controlled	RESISTANCE TRAINING MALE; RESISTANCE TRAINING FEMALE	8	8	2
Centner 2019 [54]	Achilles	Randomized parallel controlled	HIGH LOAD RESISTANCE; LOW LOAD BLOOD FLOW RESTRICTION	NE	14; 11	2
Werkhausen 2018 [53,55]	Achilles	Uncontrolled single group prepost	EXPLOSIVE ISOMETRIC PLANTAR FLEXION	10	11	1
Walker 2020 [56]	Patellar	Randomized parallel controlled	ACCENTUATED ECCENTRIC LOADING; TRADITIONAL RESISTANCE	10	10	2
Carroll 2011 [77]	Patellar	Randomized parallel controlled	KNEE EXTENSION RESISTANCE TRAINING ACETAMINOPHEN; KNEE EXTENSION RESISTANCE TRAINING IBUPROFEN; KNEE EXTENSION RESISTANCE TRAINING PLACEBO	12	7; 8	3
Epro 2017 [72]	Achilles	Uncontrolled single group prepost	High-load cyclic isometric plantar-flexion training	NE	21	1
Eriksen 2019 [70]	Patellar	Randomized parallel controlled	HEAVY RESISTANCE TRAINING; MODERATE RESISTANCE TRAINING	52	10; 13	2
Grosset 2014 [75]	Patellar	Randomized parallel controlled	HIGH INTENSITY KNEE EXTENSION RESISTANCE TRAINING; LOW INTENSITY KNEE EXTENSION RESISTANCE TRAINING	12	8; 9	2
Hirayama 2017 [73]	Achilles	Randomized parallel controlled	PLYOMETRIC DEPTH JUMP	12	11	1
Jacobs 2025 [59]	Patellar	Non-Randomized parallel controlled	MAXIMAL STRENGTH TRAINING	12	9	1
Jerger 2022 [68]	Achilles; Patellar	Randomized parallel controlled	HIGH LOAD RESISTANCE PLA; HIGH LOAD RESISTANCE SCP	14	14; 15; 13; 16	4
Lambrianides 2024 [64]	Achilles	Randomized within person	ISOMETRIC RESISTANCE LONG MTU DORSIFLEXED; ISOMETRIC RESISTANCE SHORT MTU PLANTARFLEXED	12	12	2
Laurent 2020 [69]	Achilles	Randomized parallel controlled	PLYOMETRIC TRAINING KE; PLYOMETRIC TRAINING KF	10	11	2
Lee 2023 [67]	Patellar	Randomized parallel controlled	SOCCER STRENGTH PLYOMETRIC COLLAGEN; SOCCER STRENGTH PLYOMETRIC PLACEBO	NE	8; 9	2
Lee 2025 [63]	Patellar	Randomized parallel controlled	SOCCER PLUS RESISTANCE TRAINING COLLAGEN; SOCCER PLUS RESISTANCE TRAINING PLACEBO	10	6; 5	2
Nulty 2025 [61,91]	Patellar	Randomized parallel controlled	RESISTANCE TRAINING COLLAGEN; RESISTANCE TRAINING PLACEBO	12	9; 11	2
Vesterhus 2025 [60]	Patellar	Non-Randomized parallel controlled	RESISTANCE TRAINING NOC; RESISTANCE TRAINING OC	12	17; 15	2
Balshaw 2023 [82]	Patellar	Randomized parallel controlled	LOWER BODY RESISTANCE COLLAGEN PEPTIDE; LOWER BODY RESISTANCE PLACEBO	15	19; 20	2
Bontemps 2024 [81]	Patellar	Uncontrolled single group prepost	DOWNHILL RUNNING TRAINING	4	12	1
Domroes 2024 [85]	Patellar	Non-Randomized parallel controlled	PERSONALIZED TENDON LOADING	31	12	1
Kubo 2002 [84]	Achilles	Non-Randomized within person	RESISTANCE PLUS STRETCHING; RESISTANCE TRAINING	NE	8	2
Onambele-Pearson 2012 [83]	Patellar	Uncontrolled single group prepost	PROGRESSIVE RESISTANCE TRAINING FEMALE; PROGRESSIVE RESISTANCE TRAINING MALE	12	7	2
Kearns 2026 [87,88]	Achilles	Non-Randomized parallel controlled	Endurance resistance training + plyometrics (ERPT); HEAVY RESISTANCE PLUS PLYOMETRIC; HEAVY RESISTANCE	10	8; 9	3
Quinlan 2021 [92]	Patellar	Randomized parallel controlled	OLDER CONCENTRIC; OLDER ECCENTRIC; YOUNG CONCENTRIC; YOUNG ECCENTRIC	NE	8; 9; 10	4
Albracht 2013 [43]	Achilles	Non-Randomized parallel controlled allocation method NR	Bilateral isometric plantar flexion90% MVC plus habitual running; 4/week; 5 × 4; 3 s on/off	14	13	1

Source-reported analyzed n and matched units are distinct quantities. Participant, limb, and arm fields are reported separately in Table S1. Repeated arms, outcomes, and tendon sites must not be summed to infer independent participant totals. Companion reports supplement the same biological study; Albracht 2013 [43] and Bohm 2021 [79] are distinct cohorts.

**Table 2:** Primary and key secondary meta-analysis results.

Analysis	Metric	Estimate	95% CI	CR2 df	*p*	Natural Scale	Units/Samples/Cohorts
Strength	lnRR	0.1495	0.1210 to 0.1779	34.59	<0.001	+16.12% (+12.87% to +19.47%)	70/62/37
Tendon stiffness	lnRR	0.2156	0.1638 to 0.2674	32.77	<0.001	+24.06% (+17.80% to +30.66%)	70/62/37
Strength − stiffness	delta	−0.0661	−0.1190 to −0.0132	30.81	0.016	exp(δ) = 0.9360	70/62/37
Strict maximal strain	lnRR	−0.0489	−0.1216 to 0.0239	12.36	0.170	−4.77% (−11.45% to +2.42%)	24/22/14
RoB sensitivity	delta	−0.0711	−0.1366 to −0.0057	25.42	0.034	exp(δ) = 0.9313	56/50/30
Controlled-design	delta	−0.0403	−0.0974 to 0.0169	19.56	0.157	exp(δ) = 0.9605	47/42/23
Active-adjunct exclusion	delta	−0.0729	−0.1275 to −0.0184	30.57	0.010	exp(δ) = 0.9297	62/55/37
Control-adjusted sensitivity	delta	−0.1319	−0.2511 to −0.0127	8.62	0.034	exp(δ) = 0.8764	18/18/11

CI, confidence interval; CR2, cluster-robust small-sample correction; df, degrees of freedom; lnRR, log response ratio; RoB, risk of bias. Unit/sample/cohort counts refer to the analysis shown; control-adjusted counts refer to intervention units/samples and independent cohorts, with 11 unique concurrent controls. Controlled-design restriction does not adjust for control-group changes.

## Data Availability

Analytic datasets, biological-unit mappings, selected source-provenance records, statistical outputs, and original R scripts are publicly available in Zenodo at https://doi.org/10.5281/zenodo.22769263 (accessed on 18 September 2026). The public release includes the corrected training-mode mapping, moderator outputs and audit, expanded Table S1 characteristics, recovered search instructions, and current risk-of-bias ledger. Anonymous access and download were verified; standalone execution of the complete code archive was not tested.

## Supplementary Materials

The following supporting information can be downloaded at: https://www.mdpi.com/article/10.3390/jfmk11040393/s1, Table S1, expanded study and measurement characteristics; Table S2, final moderator analyses; Table S3, control-adjusted results and comparator inventory; Table S4, retained electronic search strategies and source counts; Table S5, outcome-selection hierarchy and strain definitions; Table S6, selected excluded reports; Table S7, cohort-level and synthesis-specific risk of bias; Supplementary Statistical Methods and internal specification/change inventory; Figure S1, corrected RoB profile; Figure S2, small-study diagnostic; Figure S3, matched-unit adaptation gaps. Data, provenance and original analysis scripts are provided in the accompanying reproducibility package. Supporting methodological and study references, including the selected excluded reports in Table S6, are listed in the main reference list [19,20,21,22,23,24,25,26,27,28,29,30,31,32,33,34,35,36,37,38,39,40,41,42,43,44,45,46,47,48,49,50,51,52,53,54,55,56,57,58,59,60,61,62,63,64,65,66,67,68,69,70,71,72,73,74,75,76,77,78,79,80,81,82,83,84,85,86,87,88,89,90,91,92,93,94,95,96,97,98,99].

## Abbreviations

CI, confidence interval; CR2, bias-reduced cluster-robust variance estimator; FDR, false discovery rate; HTZ, approximate Hotelling’s T^2^-Z small-sample Wald test; lnRR, log response ratio; MVC, maximal voluntary contraction; PRISMA, Preferred Reporting Items for Systematic Reviews and Meta-Analyses; PRISMA-S, PRISMA extension for literature searches; REML, restricted maximum likelihood; RoB 2, Risk of Bias 2 tool; ROBINS-I, Risk Of Bias In Non-randomized Studies of Interventions.
