# Supplementary material for: Do Strength and Tendon Stiffness Adapt in Parallel? A Systematic Review and Multilevel Meta-Analysis of Chronic Mechanical Loading in Healthy Humans
Source: J Funct Morphol Kinesiol. 2026 Sep 29;11(4):393. doi: 10.3390/jfmk11040393 (PMC13692497; doi:10.3390/jfmk11040393)
Supplement: Supplementary file 1 [file jfmk-11-00393-s001.zip › jfmk-4556726-supplementary.pdf]

Supplementary materials

Do Strength and Tendon Stiffness Adapt in Parallel? A Systematic Review and Multilevel Meta-Analysis of Chronic Mechanical Loading in Healthy Humans

Keren Pang and Zirui Li
Department of Sports & Health Science, Shinhan University, 95 Hoam-ro, Uijeongbu-si 11644, Gyeonggi-do, Republic of Korea

This supplement connects the frozen study identities, available characteristics and corrected statistical outputs. No primary or other frozen statistical models were refitted during final document integration. Targeted source verification identified three training-mode classification corrections (U19, U20 and U32), and only the training-mode moderator analysis and the associated false-discovery-rate adjustment were updated. U63 was relabelled to reflect the source-described endurance-resistance-plus-plyometrics intervention without changing its moderator category. Primary effect sizes and all other frozen analyses remained unchanged. The demographic, loading and measurement-method backfill for Table S1 was a reporting/source-verification task and did not alter eligibility, biological identity or risk-of-bias judgments.

Contents: Table S1, study and measurement characteristics; Table S2, moderators; Table S3, control-adjusted evidence; Table S4, search records; Table S5, endpoint hierarchy and strain definitions; Supplementary Statistical Methods and specification/change inventory; Figures S1–S3. Table S6, selected excluded reports; Table S7, cohort-level and synthesis-specific risk of bias.

Table S1 Expanded study characteristics

The review-level inventory includes all 50 cohorts. Detailed unit tables cover the frozen primary set and use short U identifiers linked to the full biological keys in the accompanying CSV. Participant, limb, arm and unit counts are distinct. Do not sum repeated source n values across units, outcomes or tendon sites. NR means explicitly not reported; NE means not extracted or unavailable after the retained-record and targeted source-verification pass. No missing characteristic was inferred from an absent extraction field.

S1a Review-level cohort inventory

Study	Tendon	Design	Samples	Arms	Primary units
Reeves 2003	Patellar	Uncontrolled pre/post	1	1	1
Arampatzis 2007	Achilles	Randomized within-person	1	2	2
Kongsgaard 2007	Patellar	Randomized within-person	1	2	2
Seynnes 2009	Patellar	Uncontrolled pre/post	1	1	1
Arampatzis 2010	Achilles	Non-randomized within-person	1	2	0
Kubo 2012	Achilles	Uncontrolled pre/post	1	1	1
Malliaras 2013	Patellar	Randomized parallel	3	3	3
McMahon 2013	Patellar	Randomized parallel	3	3	3
Bohm 2014	Achilles	Non-randomized within-person	2	4	0
Kubo 2017	Achilles	Randomized within-person	1	2	2
Kubo 2017	Patellar	Randomized within-person	1	2	2
Geremia 2018	Achilles	Uncontrolled pre/post	1	1	1
Massey 2018	Patellar	Randomized parallel	2	2	2
McMahon 2018	Patellar	Non-randomized parallel	2	2	2
Waugh 2018	Achilles	Non-randomized parallel	1	2	0
Werkhausen 2018	Achilles	Uncontrolled pre/post	1	1	1
Centner 2019	Achilles	Randomized parallel	2	2	2
Walker 2020	Patellar	Randomized parallel	2	2	2
Cesanelli 2026	Patellar	Non-randomized parallel	2	2	0
Jacobs 2025	Patellar	Non-randomized parallel	1	1	1
Vesterhus 2025	Patellar	Non-randomized parallel	2	2	2
Nulty 2025	Patellar	Randomized parallel	2	2	2
Létocart 2024	Achilles;Patellar	Non-randomized parallel	3	3	0
Lee 2025	Patellar	Randomized parallel	2	2	2
Lambrianides 2024	Achilles	Randomized within-person	1	2	2

Study	Tendon	Design	Samples	Arms	Primary units
Jerger 2022	Achilles;Patellar	Randomized parallel	2	2	4
Lee 2023	Patellar	Randomized parallel	2	2	2
Laurent 2020	Achilles	Randomized parallel	2	2	2
Eriksen 2019	Patellar	Randomized parallel	2	2	2
Eriksen 2018	Patellar	Randomized parallel	2	2	0
Epro 2017	Achilles	Uncontrolled pre/post	1	1	1
Hirayama 2017	Achilles	Randomized parallel	1	1	1
Vikmoen 2016	Patellar	Non-randomized parallel	1	1	0
Grosset 2014	Patellar	Randomized parallel	2	2	2
Fouré 2013	Achilles	Randomized parallel	1	1	0
Carroll 2011	Patellar	Randomized parallel	3	3	3
Fouré 2010	Achilles	Non-randomized parallel	1	1	0
Kay 2024	Achilles	Non-randomized parallel	1	1	0
Bontemps 2024	Patellar	Uncontrolled pre/post	1	1	1
Balshaw 2023	Patellar	Randomized parallel	2	2	2
Onambele-Pearson 2012	Patellar	Uncontrolled pre/post	2	2	2
Kubo 2002	Achilles	Non-randomized within-person	1	2	2
Domroes 2024	Patellar	Non-randomized parallel	1	1	1
Tsai 2024	Achilles	Non-randomized within-person	1	5	0
Kearns 2026	Achilles	Non-randomized parallel	3	3	3
Moltubakk 2021	Achilles	Non-randomized within-person	1	2	0
Ben 2018	Achilles	Randomized parallel	2	2	0
Quinlan 2021	Patellar	Randomized parallel	4	4	4
Albracht 2013	Achilles	Non-randomized parallel; allocation NR	1	1	1
Bohm 2021	Achilles	Randomized parallel	1	1	1

S1b Demographics and measurement units

Unit / study	Sample	Participants	Limbs	Arms	Units	Age	Sex / training status
U01 Seynnes 2009	S01	15	15	1	1	20 +/- 2 y	Male; Healthy; training status not fully extracted

Unit / study	Sample	Participants	Limbs	Arms	Units	Age	Sex / training status
U02 Bohm 2021	S02	13	13	1	1	29 (5)	4F/9M; recreational runners (≥2 running sessions/week)
U03 Reeves 2003	S03	9	9	1	1	74.3 (SD 3.5)	5F/4M; Older adults; not resistance trained
U04 Arampatzis 2007	S04	11	11	1	1	29.5 (SD 5.0)	8F/3M; Healthy; not strength trained
U05 Arampatzis 2007	S04	11	11	1	1	29.5 (SD 5.0)	8F/3M; Healthy; not strength trained
U06 Kongsgaard 2007	S05	12	12	1	1	24.6 ± 1.0 y (SEM)	M; Healthy young men
U07 Kongsgaard 2007	S05	12	12	1	1	24.6 ± 1.0 y (SEM)	M; Healthy young men
U08 McMahon 2013	S06	10	NE	1	1	19 ± 2.2 y	6M/4F; physically active; no lower-limb resistance training in prior 12 months
U09 McMahon 2013	S07	11	NE	1	1	21 ± 3.4 y	5M/6F; physically active; no lower-limb resistance training in prior 12 months
U10 McMahon 2013	S08	11	NE	1	1	19 ± 2.6 y	7M/4F; physically active; no lower-limb resistance training in prior 12 months
U11 Kubo 2012	S09	9	NE	1	1	21-27	M; Healthy young men
U12 Kubo 2017	S10	11	11	1	1	22.5 +/- 3.2 y	Male; healthy; no regular exercise training
U13 Kubo 2017	S10	11	11	1	1	22.5 +/- 3.2 y	Male; healthy; no regular exercise training
U14 Malliaras 2013	S11	9	NE	1	1	18–35 y (eligibility range)	Male; healthy young; not regular weight trainers
U15 Malliaras 2013	S12	10	NE	1	1	18–35 y (eligibility range)	Male; healthy young; not regular weight trainers
U16 Malliaras 2013	S13	10	NE	1	1	18–35 y (eligibility range)	Male; healthy young; not regular weight trainers
U17 Kubo 2017	S14	7	7	1	1	20.8 (SD 0.5)	M; Physically active; no organized training in prior year

Unit / study	Sample	Participants	Limbs	Arms	Units	Age	Sex / training status
U18 Kubo 2017	S14	7	7	1	1	20.8 (SD 0.5)	M; Physically active; no organized training in prior year
U19 Massey 2018	S15	13	NE	1	1	25 ± 2 y	Male; healthy young adults; no lower-body strength training for >18 months
U20 Massey 2018	S16	15	NE	1	1	25 ± 2 y	Male; healthy young adults; no lower-body strength training for >18 months
U21 Geremia 2018	S17	15	NE	1	1	26 (SD 5)	M; Healthy physically active men
U22 McMahon 2018	S18	8	NE	1	1	20 ± 1 y	Male; recreationally active; no recent lower-limb resistance training
U23 McMahon 2018	S19	8	NE	1	1	19 ± 3 y	Female; recreationally active; no recent lower-limb resistance training
U24 Centner 2019	S20	14	NE	1	1	26.1 ± 4.2 y	Male; healthy; untrained
U25 Centner 2019	S21	11	NE	1	1	27.1 ± 4.7 y	Male; healthy; untrained
U26 Werkhausen 2018	S22	11	11	1	1	26 +/- 4 y	Mixed (5M/6F); Healthy
U27 Walker 2020	S23	10	NE	1	1	21 ± 2 y	Male; resistance-trained
U28 Walker 2020	S24	10	NE	1	1	21 ± 2 y	Male; resistance-trained
U29 Carroll 2011	S25	7	NE	1	1	64 ± 1 y (SEM)	Source arm 7M/4F; older adults (acetaminophen arm; arm-level demographics)
U30 Carroll 2011	S26	8	NE	1	1	64 ± 1 y (SEM)	Source arm 9M/4F; older adults (ibuprofen arm; arm-level demographics)
U31 Carroll 2011	S27	7	NE	1	1	67 ± 2 y (SEM)	Source arm 8M/4F; older adults (placebo arm; arm-level demographics)
U32 Epro 2017	S28	21	NE	1	1	60–75 y	Female; older adults
U33 Eriksen 2019	S29	10	NE	1	1	67 ± 2.3 y (SD)	7M/3F; untrained older adults (heavy-resistance group)
U34 Eriksen 2019	S30	13	NE	1	1	66 ± 2.4 y (SD)	5M/8F; untrained older adults (moderate-resistance group)
U35 Grosset 2014	S31	8	NE	1	1	68 ± 6 y	Mixed sex; older adults; pair-matched habitual activity

Unit / study	Sample	Participants	Limbs	Arms	Units	Age	Sex / training status
U36 Grosset 2014	S32	9	NE	1	1	74 ± 5 y	Mixed sex; older adults; pair-matched habitual activity
U37 Hirayama 2017	S33	11	11	1	1	22 ± 3 y	Male; recreationally active; no habitual plyometric exercise
U38 Jacobs 2025	S34	9	NE	1	1	26 ± 6 y	Male; well-trained triathletes (≥3 y; ≥8 h/week endurance training)
U39 Jerger 2022	S35	14	NE	1	1	26.3 ± 4.0 y	Male; healthy; no recent systematic resistance training
U40 Jerger 2022	S35	15	NE	1	1	26.3 ± 4.0 y	Male; healthy; no recent systematic resistance training
U41 Jerger 2022	S36	13	NE	1	1	26.3 ± 4.0 y	Male; healthy; no recent systematic resistance training
U42 Jerger 2022	S36	16	NE	1	1	26.3 ± 4.0 y	Male; healthy; no recent systematic resistance training
U43 Lambrianides 2024	S37	12	NE	1	1	Young adults	Mixed sex; healthy young adults
U44 Lambrianides 2024	S37	12	NE	1	1	Young adults	Mixed sex; healthy young adults
U45 Laurent 2020	S38	11	11	1	1	19–26 y	6M/5F; physically active students; no regular strength/plyometric training
U46 Laurent 2020	S39	11	11	1	1	19–26 y	6M/5F; physically active students; no regular strength/plyometric training
U47 Lee 2023	S40	8	NE	1	1	17 ± 0.9 y	Female; competitive soccer players
U48 Lee 2023	S41	9	NE	1	1	17 ± 0.9 y	Female; competitive soccer players
U49 Lee 2025	S42	6	NE	1	1	25.7 ± 4.2 y	Female; professional soccer athletes

Unit / study	Sample	Participants	Limbs	Arms	Units	Age	Sex / training status
U50 Lee 2025	S43	5	NE	1	1	25.7 ± 4.2 y	Female; professional soccer athletes
U51 Nulty 2025	S44	9	NE	1	1	47 ± 5 y	Male; recreationally active
U52 Nulty 2025	S45	11	NE	1	1	47 ± 5 y	Male; recreationally active
U53 Vesterhus 2025	S46	17	NE	1	1	31.6 ± 4.8 y	Female; eumenorrheic non-OC users; young/untrained
U54 Vesterhus 2025	S47	15	NE	1	1	27.9 ± 2.8 y	Female; combined oral-contraceptive users; young/untrained
U55 Balshaw 2023	S48	19	19	1	1	27.0 ± 5.0 y	Male; young healthy; recreationally active
U56 Balshaw 2023	S49	20	20	1	1	24.4 ± 3.2 y	Male; young healthy; recreationally active
U57 Bontemps 2024	S50	12	NE	1	1	Young adults	5F/7M; untrained healthy young adults
U58 Domroes 2024	S51	12	NE	1	1	15–16 y	Male; adolescent elite handball athletes
U59 Kubo 2002	S52	8	8	1	1	21 ± 2 y	Male; healthy; recreational sports; no regular strength/flexibility training
U60 Kubo 2002	S52	8	8	1	1	21 ± 2 y	Male; healthy; recreational sports; no regular strength/flexibility training
U61 Onambele-Pearson 2012	S53	7	NE	1	1	76.7 ± 1.2 y (SEM)	Female; habitually active older adults
U62 Onambele-Pearson 2012	S54	7	NE	1	1	74.0 ± 1.2 y (SEM)	Male; habitually active older adults
U63 Kearns 2026	S55	8	NE	1	1	51 ± 5 y	Mixed sex; recreational middle-aged runners

Unit / study	Sample	Participants	Limbs	Arms	Units	Age	Sex / training status
U64 Kearns 2026	S56	9	NE	1	1	51 ± 5 y	Mixed sex; recreational middle-aged runners
U65 Kearns 2026	S57	9	NE	1	1	51 ± 5 y	Mixed sex; recreational middle-aged runners
U66 Quinlan 2021	S58	8	8	1	1	69.1 ± 3.0 y	Male; healthy older adults
U67 Quinlan 2021	S59	9	9	1	1	67.5 ± 1.5 y	Male; healthy older adults
U68 Quinlan 2021	S60	10	10	1	1	23.3 ± 3.8 y	Male; healthy young adults
U69 Quinlan 2021	S61	10	10	1	1	25.3 ± 6.2 y	Male; healthy young adults
U70 Albracht 2013	S62	13	13	1	1	27 (5)	Sex not specified in accessible participant paragraph; recreational long-distance runners (≥3 sessions/week, 30–120 km/week)

S1c Loading and comparators

Unit	Intervention / weeks	Frequency	Intensity	Volume	Comparator
U01	HEAVY KNEE EXTENSION RESISTANCE TRAINING; 9 weeks	3/week	80% 1RM (warm-up 40%)	4 × 10 knee extensions; 2-min rest; 1RM adjusted weekly	No concurrent control in primary unit
U02	Isometric plantar flexion90% MVC plus habitual running;3-4/week;5x4;3s on/off; supervised; 14 weeks	3–4/week	≈90% MVC	5 × 4 isometric plantar-flexion reps; 3 s loading / 3 s relaxation	Habitual running; matched control F+K available
U03	HEAVY RESISTANCE; 14 weeks	3/week	80% of 5RM	2 × 10 leg extension + leg press	Non-training control
U04	LOW STRAIN; 14 weeks	4/week	55% MVC	5 × 7 isometric plantar-flexion reps; 3 s loading / 3 s relaxation	Contralateral high-strain leg (within-person)

Unit	Intervention / weeks	Frequency	Intensity	Volume	Comparator
U05	HIGH STRAIN; 14 weeks	4/week	90% MVC	5 × 4 isometric plantar-flexion reps; 3 s loading / 3 s relaxation	Contralateral low-strain leg (within-person)
U06	LIGHT RESISTANCE; 12 weeks	3/week	Light load; work matched to heavy leg	10 × 36 knee extensions; 30-s set rest	Contralateral heavy-resistance leg (within-person)
U07	HEAVY RESISTANCE; 12 weeks	3/week	70% 1RM	10 × 8 knee extensions; 3-min set rest	Contralateral light-resistance leg (within-person)
U08	RESISTANCE TRAINING MTCS; 8 weeks	3/week	80% 1RM	Weeks 1–4: 3 × 10; weeks 5–8: 4 × 8; four lower-body exercises/session	Non-training habitual-activity control
U09	RESISTANCE TRAINING MTCL; 8 weeks	3/week	55% 1RM	Weeks 1–4: 3 × 10; weeks 5–8: 4 × 8; four lower-body exercises/session	Non-training habitual-activity control
U10	RESISTANCE TRAINING MTCX; 8 weeks	3/week	80% 1RM	Weeks 1–4: 3 × 10; weeks 5–8: 4 × 8; four lower-body exercises/session	Non-training habitual-activity control
U11	ISOMETRIC PLANTAR FLEXION TRAINING; 12 weeks	4/week	80% MVC	15 × 15-s isometric plantar-flexion contractions/session	No concurrent control during the 12-week training phase; detraining followed training
U12	PLYOMETRIC; 12 weeks	3/week	Unilateral plantar-flexion / jump loading; ~40% 1RM external load for the plyometric protocol	5 sets hopping + 5 sets drop jumps; 10 reps/set; 30-s rests	Contralateral isometric leg (within-person)
U13	ISOMETRIC; 12 weeks	3/week	80% MVC	10 × 15-s isometric contractions; 30-s rests	Contralateral plyometric leg (within-person)

Unit	Intervention / weeks	Frequency	Intensity	Volume	Comparator
U14	CONCENTRIC; 12 weeks	3/week	80% concentric–eccentric 1RM	4 × 7–8 concentric repetitions	Non-training control
U15	STANDARD ECCENTRIC; 12 weeks	3/week	80% concentric–eccentric 1RM	4 × 12–15 eccentric repetitions	Non-training control
U16	HIGH LOAD ECCENTRIC; 12 weeks	3/week	80% eccentric 1RM	4 × 7–8 eccentric repetitions	Non-training control
U17	CONCENTRIC; 12 weeks	3/week	80% 1RM	5 × 10 unilateral knee extensions; ~1 s concentric; 1-min rest	Contralateral eccentric leg (within-person)
U18	ECCENTRIC; 12 weeks	3/week	80% 1RM	5 × 10 unilateral knee extensions; ~3 s eccentric; 1-min rest	Contralateral concentric leg (within-person)
U19	Explosive-contraction isometric knee-extension strength training (ECT); 12 weeks	3/week	Rapid explosive effort to ~80% MVT	40 isometric knee-extension contractions/session	Non-training control
U20	Sustained-contraction isometric knee-extension strength training (SCT); 12 weeks	3/week	Gradually to ~75% MVT; sustained hold	40 isometric knee-extension contractions/session; ~3 s sustained phase	Non-training control
U21	HIGH LOAD ECCENTRIC PLANTAR FLEXION TRAINING; 12 weeks	23 sessions over 12 weeks (generally 2/week)	Maximal eccentric plantar-flexor contractions at 30°/s	Weeks 1–4: 3 × 10; weeks 5–8: 4 × 10; weeks 9–12: 5 × 10	No concurrent external control; a 4-week pre-training no-intervention control period preceded training
U22	RESISTANCE TRAINING MALE; 8 weeks	3/week	~80% 1RM	Weeks 1–4: 3 × 10; weeks 5–8: 4 × 8; lower-body exercises	Sex-stratified training group; separate non-training controls in source study
U23	RESISTANCE TRAINING FEMALE; 8 weeks	3/week	~80% 1RM	Weeks 1–4: 3 × 10; weeks 5–8: 4 × 8; lower-body exercises	Sex-stratified training group; separate non-training controls in source study

Unit	Intervention / weeks	Frequency	Intensity	Volume	Comparator
U24	HIGH LOAD RESISTANCE; 14 weeks	3/week	70–85% 1RM	High-load calf-raise resistance training	Non-exercising control
U25	LOW LOAD BLOOD FLOW RESTRICTION; 14 weeks	3/week	20–35% 1RM with blood-flow restriction	Low-load calf-raise resistance training with BFR	Non-exercising control
U26	EXPLOSIVE ISOMETRIC PLANTAR FLEXION; 10 weeks	3/week	Explosive maximal-intent isometric plantar flexion; target ~80% weekly maximum force	4 × 10 unilateral contractions; 1 s contraction; 5 s rest	Non-training control
U27	ACCENTUATED ECCENTRIC LOADING; 10 weeks	2/week	6RM session + 40% eccentric overload; 10RM session + 40% eccentric overload	3 sets per exercise/session; leg press, knee extension, knee flexion	Habitual strength-training control
U28	TRADITIONAL RESISTANCE; 10 weeks	2/week	6RM and 10RM sessions; same concentric/eccentric load	3 sets per exercise/session; leg press, knee extension, knee flexion	Habitual strength-training control
U29	KNEE EXTENSION RESISTANCE TRAINING ACETAMINOPHEN; 12 weeks	3/week	Mean training load ≈71% 1RM (acetaminophen arm)	3 × 10 bilateral knee extensions; load progressed from 1RM testing	Same resistance training + placebo/ibuprofen comparator arms
U30	KNEE EXTENSION RESISTANCE TRAINING IBUPROFEN; 12 weeks	3/week	Mean training load ≈73% 1RM (ibuprofen arm)	3 × 10 bilateral knee extensions; load progressed from 1RM testing	Same resistance training + placebo/acetaminophen comparator arms

Unit	Intervention / weeks	Frequency	Intensity	Volume	Comparator
U31	KNEE EXTENSION RESISTANCE TRAINING PLACEBO; 12 weeks	3/week	Mean training load ≈74% 1RM (placebo arm)	3 × 10 bilateral knee extensions; load progressed from 1RM testing	Same resistance training + acetaminophen/ibuprofen comparator arms
U32	High-load cyclic isometric plantar-flexion training; 14 weeks	3/week (2/week during weeks 1–2)	≈90% MVC	5 × 4 contractions with each leg individually and bilaterally; 3 s loading / 3 s relaxation; ~50 min/session	Concurrent non-training/usual-activity control (n=13)
U33	HEAVY RESISTANCE TRAINING; 52 weeks	3/week	70–90% 1RM	3–5 sets; leg press + knee extension within whole-body HRT	Usual-activity control
U34	MODERATE RESISTANCE TRAINING; 52 weeks	3/week	Moderate-load elastic/body-weight circuit; load progressed with capacity	3 × 12 progressing to 3 × 18; squat + knee extension among whole-body circuit	Usual-activity control
U35	HIGH INTENSITY KNEE EXTENSION RESISTANCE TRAINING; 12 weeks	3/week	~80% 1RM	2–4 sets × 8–11 reps; supervised cable resistance plus home-band sessions; 3 sessions/week total	Low-intensity active resistance-training comparator
U36	LOW INTENSITY KNEE EXTENSION RESISTANCE TRAINING; 12 weeks	3/week	~40% 1RM	2–4 sets × 8–11 reps; supervised cable resistance plus home-band sessions; 3 sessions/week total	High-intensity active resistance-training comparator
U37	PLYOMETRIC DEPTH JUMP; 12 weeks	3/week	Body/sledge depth-jump loading; no fixed %1RM/MVC prescription	10 × 10 depth jumps; 30 s rest; 1-week mid-program rest	Non-training control
U38	MAXIMAL STRENGTH TRAINING; 12 weeks	3/week	~80–90% 1RM	Five strength exercises; 3–10 reps/set; ~3-min rests; ~1 s concentric / 2 s eccentric	Endurance-only control

Unit	Intervention / weeks	Frequency	Intensity	Volume	Comparator
U39	HIGH LOAD RESISTANCE PLA; 14 weeks	3/week	Progressive 70→85% 1RM	Seated + standing calf raises; 3 sets each; 12→6 reps; ~4 s TUT	Same high-load training + specific collagen peptide arm
U40	HIGH LOAD RESISTANCE PLA; 14 weeks	3/week	Progressive 70→85% 1RM	Seated + standing calf raises; 3 sets each; 12→6 reps; ~4 s TUT	Same high-load training + specific collagen peptide arm
U41	HIGH LOAD RESISTANCE SCP; 14 weeks	3/week	Progressive 70→85% 1RM	Seated + standing calf raises; 3 sets each; 12→6 reps; ~4 s TUT	Same high-load training + placebo arm
U42	HIGH LOAD RESISTANCE SCP; 14 weeks	3/week	Progressive 70→85% 1RM	Seated + standing calf raises; 3 sets each; 12→6 reps; ~4 s TUT	Same high-load training + placebo arm
U43	ISOMETRIC RESISTANCE LONG MTU DORSIFLEXED; 12 weeks	3/week	80% MVC	3 sets of 3 s loading / 3 s unloading contractions to failure	Contralateral short-MTU leg (within-person)
U44	ISOMETRIC RESISTANCE SHORT MTU PLANTARFLEXED; 12 weeks	3/week	80% MVC	3 sets of 3 s loading / 3 s unloading contractions to failure	Contralateral long-MTU leg (within-person)
U45	PLYOMETRIC TRAINING KE; 10 weeks	2/week	Bodyweight hopping/drop-jump loading; no fixed %1RM/MVC prescription	6–8 exercises; 3–5 × 10; progression ~200→400 jumps/session; ~1.5-min set and ~3-min exercise rests	Concurrent non-training control (n=10) maintained normal daily activity
U46	PLYOMETRIC TRAINING KF; 10 weeks	2/week	Bodyweight hopping/drop-jump loading; no fixed %1RM/MVC prescription	6–8 exercises; 3–5 × 10; progression ~200→400 jumps/session; ~1.5-min set and ~3-min exercise rests	Concurrent non-training control (n=10) maintained normal daily activity
U47	SOCCER STRENGTH PLYOMETRIC COLLAGEN; 10 weeks	3 supplementation/training days/week	Mixed bodyweight strength/plyometric/pitch-based loading; no single %1RM/MVC prescription	In-season soccer + bodyweight strength/plyometric/pitch-based exercise	Same soccer/training + placebo

Unit	Intervention / weeks	Frequency	Intensity	Volume	Comparator
U48	SOCCER STRENGTH PLYOMETRIC PLACEBO; 10 weeks	3 supplementation/training days/week	Mixed bodyweight strength/plyometric/pitch-based loading; no single %1RM/MVC prescription	In-season soccer + bodyweight strength/plyometric/pitch-based exercise	Same soccer/training + collagen
U49	SOCCER PLUS RESISTANCE TRAINING COLLAGEN; 10 weeks	3/week	High-intensity lower-limb training	Resistance, plyometric or pitch-based exercise; 30 g collagen + 500 mg vitamin C before training	Same soccer/training + placebo
U50	SOCCER PLUS RESISTANCE TRAINING PLACEBO; 10 weeks	3/week	High-intensity lower-limb training	Resistance, plyometric or pitch-based exercise; energy-matched placebo + 500 mg vitamin C	Same soccer/training + collagen
U51	RESISTANCE TRAINING COLLAGEN; 12 weeks	2/week	Started at ~90% 10RM; progressive loading	Weeks 1–6: 4 × 8–10; weeks 7–12: 4 × 6–8; 3-min rests; +2.5–5 kg after completing top rep target	Same resistance training + placebo
U52	RESISTANCE TRAINING PLACEBO; 12 weeks	2/week	Started at ~90% 10RM; progressive loading	Weeks 1–6: 4 × 8–10; weeks 7–12: 4 × 6–8; 3-min rests; +2.5–5 kg after completing top rep target	Same resistance training + collagen
U53	RESISTANCE TRAINING NOC; 12 weeks	24 sessions across 3 menstrual/pill cycles	Progressive RPE 7/RIR3 → RPE 10/RIR0	Heavy knee-extensor resistance training; ≥48 h between sessions	OC-user training group
U54	RESISTANCE TRAINING OC; 12 weeks	24 sessions across 3 menstrual/pill cycles	Progressive RPE 7/RIR3 → RPE 10/RIR0	Heavy knee-extensor resistance training; ≥48 h between sessions	Non-OC training group

Unit	Intervention / weeks	Frequency	Intensity	Volume	Comparator
U55	LOWER BODY RESISTANCE COLLAGEN PEPTIDE; 15 weeks	3/week	Undulating ~12RM to ~6RM	2–4 sets of knee extension, knee flexion and leg press; 2-min set rest	Same training + placebo
U56	LOWER BODY RESISTANCE PLACEBO; 15 weeks	3/week	Undulating ~12RM to ~6RM	2–4 sets of knee extension, knee flexion and leg press; 2-min set rest	Same training + collagen peptides
U57	DOWNHILL RUNNING TRAINING; 4 weeks	10 sessions over 4 weeks	Downhill running at 60–65% VO2max	Session duration progressed 15→30 min	No concurrent control
U58	PERSONALIZED TENDON LOADING; 31 weeks	3/week	Personalized loading targeting ~6.2% tendon strain	Tendon exercises integrated into usual handball training for ~31 weeks	Usual training control
U59	RESISTANCE PLUS STRETCHING; 8 weeks	Resistance 4/week; stretching 7/week	70% 1RM plantar flexion	Resistance 5 × 10; plus static stretching 10 min/day	Contralateral resistance-only leg (within-person)
U60	RESISTANCE TRAINING; 8 weeks	4/week	70% 1RM plantar flexion	5 × 10 unilateral plantar flexion	Contralateral resistance+stretching leg (within-person)
U61	PROGRESSIVE RESISTANCE TRAINING FEMALE; 12 weeks	2/week (1 supervised + 1 home session)	8RM for supervised cable exercises; loads revised every 4 weeks	Supervised cable work: 2 × 8 per exercise + home-band/walking programme	Male training stratum; no external control
U62	PROGRESSIVE RESISTANCE TRAINING MALE; 12 weeks	2/week (1 supervised + 1 home session)	8RM for supervised cable exercises; loads revised every 4 weeks	Supervised cable work: 2 × 8 per exercise + home-band/walking programme	Female training stratum; no external control

Unit	Intervention / weeks	Frequency	Intensity	Volume	Comparator
U63	Endurance resistance training + plyometrics (ERPT); 10 weeks	2 virtual sessions/week	Self-selected endurance-resistance load; plyometric exercises	1–2 sets of 10–20 repetitions; lower-limb resistance exercises and plyometrics; at least 1-min rests (Appendix A)	Alternative runner resistance-training programs
U64	HEAVY RESISTANCE PLUS PLYOMETRIC; 10 weeks	2 virtual sessions/week	Heavy resistance: self-selected challenging load for 5–8 reps; plus bodyweight plyometrics	4 × 5–8 heavy-resistance reps + 1–2 × 4–8 plyometric reps; ≥1-min rests; progressive/deload cycles	Alternative runner resistance-training programs
U65	HEAVY RESISTANCE; 10 weeks	2 virtual sessions/week	Heavy resistance: self-selected challenging load for 5–8 reps	4 × 5–8 heavy-resistance reps; ≥1-min rests; progressive/deload cycles	Alternative runner resistance-training programs
U66	OLDER CONCENTRIC; 8 weeks	3/week	60% 1RM	4 × 15; 3 s concentric contractions; 2-min rest	Age-matched eccentric training arm
U67	OLDER ECCENTRIC; 8 weeks	3/week	60% 1RM	4 × 15; 3 s eccentric contractions; 2-min rest	Age-matched concentric training arm
U68	YOUNG CONCENTRIC; 8 weeks	3/week	60% 1RM	4 × 15; 3 s concentric contractions; 2-min rest	Age-matched eccentric training arm
U69	YOUNG ECCENTRIC; 8 weeks	3/week	60% 1RM	4 × 15; 3 s eccentric contractions; 2-min rest	Age-matched concentric training arm
U70	Bilateral isometric plantar flexion90% MVC plus habitual running;4/week;5x4;3s on/off; 14 weeks	4/week	≈90% MVC	5 × 4 isometric plantar-flexion reps; 3 s loading / 3 s relaxation	No matched control F+K; control maintained habitual running

S1d Tendon mechanical methods

Unit / site	Force estimation / moment arm	Elongation method	Stiffness fitting range

Unit / site	Force estimation / moment arm	Elongation method	Stiffness fitting range
U01 / Patellar	Quadriceps force derived from corrected knee-extension torque and the study patellar-tendon moment arm.; Study-specific patellar-tendon moment arm used for tendon-force estimation.	Ultrasound patellar-tendon elongation during a maximal ramp isometric knee extension.	Highest 20% common absolute tendon-force interval.
U02 / Achilles	Joint moment divided by individual tendon lever arm.; Individual tendon-excursion lever arm.	Ultrasound tracking of gastrocnemius medialis myotendinous junction (Esaote MyLab60; source method).	50-100% maximal tendon force; resting-length normalized
U03 / Patellar	Patellar tendon force from net knee-extension torque + estimated antagonist torque divided by MRI-derived patellar-tendon moment arm.	B-mode ultrasound tracking of patellar apex displacement during ramp isometric knee extension.	Gradient of force–elongation relation by 10% force intervals; primary high-force interval 90–100% maximal tendon force.
U04 / Achilles	Plantar-flexion joint moment divided by individual Achilles tendon moment arm.	Ultrasound tracking of distal gastrocnemius medialis tendon/aponeurosis displacement during maximal plantar flexion.	High-force tendon force-elongation slope reported by the study.
U05 / Achilles	Plantar-flexion joint moment divided by individual Achilles tendon moment arm.	Ultrasound tracking of distal gastrocnemius medialis tendon/aponeurosis displacement during maximal plantar flexion.	High-force tendon force-elongation slope reported by the study.
U06 / Patellar	Knee-extension torque converted to patellar tendon force; Individual patellar tendon moment arm	Ultrasound	Maximum force-elongation stiffness; common-force analysis reported separately
U07 / Patellar	Knee-extension torque converted to patellar tendon force; Individual patellar tendon moment arm	Ultrasound	Maximum force-elongation stiffness; common-force analysis reported separately
U08 / Patellar	Corrected knee torque divided by individual patellar moment arm; DEXA-derived individual patellar moment arm	Ultrasound	Average across normalized force range; standardized 2000-N sensitivity also reported
U09 / Patellar	Corrected knee torque divided by individual patellar moment arm; DEXA-derived individual patellar moment arm	Ultrasound	Average across normalized force range; standardized 2000-N sensitivity also reported
U10 / Patellar	Corrected knee torque divided by individual patellar moment arm; DEXA-derived individual patellar moment arm	Ultrasound	Average across normalized force range; standardized 2000-N sensitivity also reported
U11 / Achilles	Study-reported Achilles tendon force-elongation assessment during plantar-flexion MVC.; Study Achilles-tendon moment-arm method.	Ultrasound Achilles tendon elongation during plantar-flexion MVC.	Study-reported high-force Achilles tendon force-elongation slope.
U12 / Achilles	Torque divided by estimated moment arm scaled to leg length; Leg-length-scaled estimate	Ultrasound of outer tendon plus aponeurosis	Slope above 50% MVC

Unit / site	Force estimation / moment arm	Elongation method	Stiffness fitting range
U13 / Achilles	Torque divided by estimated moment arm scaled to leg length; Leg-length-scaled estimate	Ultrasound of outer tendon plus aponeurosis	Slope above 50% MVC
U14 / Patellar	Knee-extension torque corrected for antagonist activity and divided by patellar tendon moment arm; Fixed 44.7 mm	Ultrasound	75-100% of maximum tendon force
U15 / Patellar	Knee-extension torque corrected for antagonist activity and divided by patellar tendon moment arm; Fixed 44.7 mm	Ultrasound	75-100% of maximum tendon force
U16 / Patellar	Knee-extension torque corrected for antagonist activity and divided by patellar tendon moment arm; Fixed 44.7 mm	Ultrasound	75-100% of maximum tendon force
U17 / Patellar	Knee-extension torque converted to patellar tendon force using the study tendon moment-arm method.	Ultrasound tracking during maximal/ramped knee extension.	Study-reported patellar tendon force-elongation stiffness.
U18 / Patellar	Knee-extension torque converted to patellar tendon force using the study tendon moment-arm method.	Ultrasound tracking during maximal/ramped knee extension.	Study-reported patellar tendon force-elongation stiffness.
U19 / Patellar	Corrected torque divided by MRI moment arm; MRI	Ultrasound	Patellar-tendon force–elongation stiffness over a standardized/pre-training force range (study primary estimate).
U20 / Patellar	Corrected torque divided by MRI moment arm; MRI	Ultrasound	Patellar-tendon force–elongation stiffness over a standardized/pre-training force range (study primary estimate).
U21 / Achilles	Corrected plantar-flexor torque divided by the individual Achilles tendon moment arm.; Individual ultrasound-derived Achilles tendon moment arm.	Ultrasound gastrocnemius medialis myotendinous-junction displacement during ramp MVIC.	Common absolute force range 825-1625 N (50-100% peak force of the weakest participant).
U22 / Patellar	Knee-extension mechanics converted to patellar tendon force using the study moment-arm procedure.	Ultrasound	Mean instantaneous stiffness averaged over 10–100% MVC
U23 / Patellar	Knee-extension mechanics converted to patellar tendon force using the study moment-arm procedure.	Ultrasound	Mean instantaneous stiffness averaged over 10–100% MVC

Unit / site	Force estimation / moment arm	Elongation method	Stiffness fitting range
U24 / Achilles	Plantar-flexion torque converted to Achilles tendon force using the study Achilles moment-arm method.	Ultrasound-based Achilles tendon elongation during plantar-flexion loading.	Calculated at the individual maximal torque level.
U25 / Achilles	Plantar-flexion torque converted to Achilles tendon force using the study Achilles moment-arm method.	Ultrasound-based Achilles tendon elongation during plantar-flexion loading.	Calculated at the individual maximal torque level.
U26 / Achilles	Torque divided by internal moment arm with 91% triceps-surae contribution; External tape measure	Ultrasound	50-80% of individual maximum tendon force
U27 / Patellar	Knee torque divided by estimated patellar moment arm; Bakenecker estimate	Ultrasound tracking of patella and tibia landmarks	50-100% of the pre-training absolute torque range
U28 / Patellar	Knee torque divided by estimated patellar moment arm; Bakenecker estimate	Ultrasound tracking of patella and tibia landmarks	50-100% of the pre-training absolute torque range
U29 / Patellar	Patellar tendon force derived from knee-extensor force/torque with study moment-arm and antagonist corrections.; Study-specific patellar-tendon moment arm.	Ultrasound tracking of patella and tibial insertion displacement during maximal contraction.	Common absolute force normalized to each group's pretraining peak tendon force.
U30 / Patellar	Patellar tendon force derived from knee-extensor force/torque with study moment-arm and antagonist corrections.; Study-specific patellar-tendon moment arm.	Ultrasound tracking of patella and tibial insertion displacement during maximal contraction.	Common absolute force normalized to each group's pretraining peak tendon force.
U31 / Patellar	Patellar tendon force derived from knee-extensor force/torque with study moment-arm and antagonist corrections.; Study-specific patellar-tendon moment arm.	Ultrasound tracking of patella and tibial insertion displacement during maximal contraction.	Common absolute force normalized to each group's pretraining peak tendon force.
U32 / Achilles	Inverse-dynamics ankle moment used as matched maximal strength endpoint; individual Achilles tendon moment arm estimated using tendon excursion	Ultrasound-based Achilles tendon elongation during maximal/loading contractions.	Study-reported high-force Achilles tendon stiffness.
U33 / Patellar	Knee-extension mechanics converted to maximal patellar tendon force; patellar-tendon moment arm estimated as 6.5% of femur length	Ultrasound	Maximal stiffness from the final 10% of the force–deformation curve
U34 / Patellar	Knee-extension mechanics converted to maximal patellar tendon force; patellar-tendon moment arm estimated as 6.5% of femur length	Ultrasound	Maximal stiffness from the final 10% of the force–deformation curve
U35 / Patellar	Corrected knee-extensor torque divided by MRI-measured patellar-tendon moment arm.; Individual MRI patellar-tendon moment arm.	B-mode ultrasound patellar-tendon elongation during ramped isometric knee extension.	Linear force-elongation fit over 60-100% MVC.
U36 / Patellar	Corrected knee-extensor torque divided by MRI-measured patellar-tendon moment arm.; Individual MRI patellar-tendon moment arm.	B-mode ultrasound patellar-tendon elongation during ramped isometric knee extension.	Linear force-elongation fit over 60-100% MVC.

Unit / site	Force estimation / moment arm	Elongation method	Stiffness fitting range
U37 / Achilles	Plantar-flexion torque divided by Achilles moment arm; MRI center-of-rotation method	Ultrasound	Study-reported Achilles tendon force–elongation stiffness during maximal loading.
U38 / Patellar	Knee-extension mechanics converted to patellar tendon force; Anthropometric approximation	Ultrasound	50-80% of fitted force-elongation curve
U39 / Achilles	Plantar-flexion torque divided by individual Achilles tendon moment arm; Individual moment arm	Ultrasound	50-80% of baseline MVC force
U40 / Patellar	Knee-extension torque divided by MRI-derived patellar tendon moment arm; Individual MRI moment arm	Ultrasound	50-80% of baseline MVC force
U41 / Achilles	Plantar-flexion torque divided by individual Achilles tendon moment arm; Individual moment arm	Ultrasound	50-80% of baseline MVC force
U42 / Patellar	Knee-extension torque divided by MRI-derived patellar tendon moment arm; Individual MRI moment arm	Ultrasound	50-80% of baseline MVC force
U43 / Achilles	Plantar-flexion torque converted to Achilles tendon force; MRI-derived Achilles tendon moment arm	Ultrasound tracking of gastrocnemius medialis muscle-tendon junction	30-80% maximal tendon force
U44 / Achilles	Plantar-flexion torque converted to Achilles tendon force; MRI-derived Achilles tendon moment arm	Ultrasound tracking of gastrocnemius medialis muscle-tendon junction	30-80% maximal tendon force
U45 / Achilles	Torque-based stiffness index; no conversion to tendon force; moment arm not applicable	Ultrasound tracking of gastrocnemius medialis muscle-tendon junction	Torque-elongation slope over 20-80% MVC
U46 / Achilles	Torque-based stiffness index; no conversion to tendon force; moment arm not applicable	Ultrasound tracking of gastrocnemius medialis muscle-tendon junction	Torque-elongation slope over 20-80% MVC
U47 / Patellar	Net knee-extension torque divided by patellar tendon moment arm; moment arm estimated from participant femur length	Ultrasound-based patellar tendon force–elongation tracking.	Final 20% tendon-force interval (80-100% of lowest absolute RMVC force).
U48 / Patellar	Net knee-extension torque divided by patellar tendon moment arm; moment arm estimated from participant femur length	Ultrasound-based patellar tendon force–elongation tracking.	Final 20% tendon-force interval (80-100% of lowest absolute RMVC force).
U49 / Patellar	Knee-extension torque converted to patellar tendon force; patellar-tendon moment arm estimated from participant femur length	Study-reported ultrasound force-elongation tracking	Study-reported primary patellar-tendon stiffness estimate during ramp isometric knee extension.
U50 / Patellar	Knee-extension torque converted to patellar tendon force; patellar-tendon moment arm estimated from participant femur length	Study-reported ultrasound force-elongation tracking	Study-reported primary patellar-tendon stiffness estimate during ramp isometric knee extension.

Unit / site	Force estimation / moment arm	Elongation method	Stiffness fitting range
U51 / Patellar	Knee-extension torque divided by patellar tendon moment arm; literature-derived patellar-tendon moment arm at 90° knee flexion	Semi-automated ultrasound tracking	Highest 20% absolute force of the weakest ramp MVC
U52 / Patellar	Knee-extension torque divided by patellar tendon moment arm; literature-derived patellar-tendon moment arm at 90° knee flexion	Semi-automated ultrasound tracking	Highest 20% absolute force of the weakest ramp MVC
U53 / Patellar	Knee-extension moment and external moment arm; quadriceps force divided by estimated internal moment arm; Estimated internal moment arm	Ultrasound force-elongation tracking	Slope over 60-100% of the lower maximum tendon force
U54 / Patellar	Knee-extension moment and external moment arm; quadriceps force divided by estimated internal moment arm; Estimated internal moment arm	Ultrasound force-elongation tracking	Slope over 60-100% of the lower maximum tendon force
U55 / Patellar	Knee-extension torque converted to patellar tendon force; Individual MRI moment arm	Ultrasound	Primary stiffness estimate reported in tendon Table 1
U56 / Patellar	Knee-extension torque converted to patellar tendon force; Individual MRI moment arm	Ultrasound	Primary stiffness estimate reported in tendon Table 1
U57 / Patellar	Net ramp-MVC knee-extension torque divided by patellar tendon moment arm with antagonist correction; Study-specific patellar tendon moment arm (0.048 m)	Ultrasound tracking during ramp MVC	Final 20% of each participant's lowest absolute ramp-MVC force
U58 / Patellar	Knee-extensor mechanics converted to patellar tendon force using study-specific tendon mechanics.	Ultrasound force-elongation tracking	Personalized tendon-loading study primary force–elongation stiffness estimate.
U59 / Achilles	Plantar-flexion torque/tendon-structure mechanics assessed during ramp isometric plantar flexion.	Ultrasonography of medial gastrocnemius tendon/aponeurosis during ramp isometric plantar flexion.	50-100% MVC force region.
U60 / Achilles	Plantar-flexion torque/tendon-structure mechanics assessed during ramp isometric plantar flexion.	Ultrasonography of medial gastrocnemius tendon/aponeurosis during ramp isometric plantar flexion.	50-100% MVC force region.
U61 / Patellar	Quadriceps maximum voluntary isometric force with study patellar-tendon mechanics protocol.; Study patellar-tendon moment-arm method.	B-mode ultrasound patellar-tendon elongation during ramped knee-extension MVC.	Common absolute tendon force of 1200 N.
U62 / Patellar	Quadriceps maximum voluntary isometric force with study patellar-tendon mechanics protocol.; Study patellar-tendon moment-arm method.	B-mode ultrasound patellar-tendon elongation during ramped knee-extension MVC.	Common absolute tendon force of 1200 N.
U63 / Achilles	Plantar-flexion MVC torque converted to Achilles tendon force using an externally measured Achilles tendon lever arm	Ultrasound	Primary Achilles force-elongation stiffness estimate

Unit / site	Force estimation / moment arm	Elongation method	Stiffness fitting range
U64 / Achilles	Plantar-flexion MVC torque converted to Achilles tendon force using an externally measured Achilles tendon lever arm	Ultrasound	Primary Achilles force-elongation stiffness estimate
U65 / Achilles	Plantar-flexion MVC torque converted to Achilles tendon force using an externally measured Achilles tendon lever arm	Ultrasound	Primary Achilles force-elongation stiffness estimate
U66 / Patellar	True knee-extensor torque divided by estimated patellar tendon moment arm; study-referenced estimation method	Ultrasound-based patellar tendon elongation during maximal/ramped knee extension.	Gradient over the final 10% of maximal patellar tendon force.
U67 / Patellar	True knee-extensor torque divided by estimated patellar tendon moment arm; study-referenced estimation method	Ultrasound-based patellar tendon elongation during maximal/ramped knee extension.	Gradient over the final 10% of maximal patellar tendon force.
U68 / Patellar	True knee-extensor torque divided by estimated patellar tendon moment arm; study-referenced estimation method	Ultrasound-based patellar tendon elongation during maximal/ramped knee extension.	Gradient over the final 10% of maximal patellar tendon force.
U69 / Patellar	True knee-extensor torque divided by estimated patellar tendon moment arm; study-referenced estimation method	Ultrasound-based patellar tendon elongation during maximal/ramped knee extension.	Gradient over the final 10% of maximal patellar tendon force.
U70 / Achilles	Maximal Achilles tendon force derived from plantar-flexion mechanics using literature-derived moment-arm data.	Ultrasound tracking of distal gastrocnemius medialis aponeurosis/tendon point (Aloka SSD4000).	50-100% maximal tendon force

The expanded_primary_unit_characteristics_FINAL.csv retains the full cohort, participant-sample, arm and report IDs for every U identifier, source locations and additional recorded fields. S aliases here are display labels only and do not create new biological identities. Previously unextracted demographic, loading and measurement fields were selectively backfilled from accessible source reports for the revision; residual NE entries indicate fields that could not be verified from the retained/accessed source materials.

Table S2 Final moderator analyses

Counts and estimates below are copied from the final frozen moderator outputs. A formal category required at least eight matched units and five cohorts. Reference levels were patellar tendon, dynamic resistance and individualized relative/maximal fitting range. Cohorts may contribute to more than one category; category counts are not additive.

Moderator	Category	Units	Cohorts	Reference	Formal
TENDON SITE	PATELLAR	46	23	TRUE	TRUE
TENDON SITE	ACHILLES	24	15	FALSE	TRUE
TRAINING MODE	DYNAMIC RESISTANCE	40	22	TRUE	TRUE
TRAINING MODE	ISOMETRIC	10	8	FALSE	TRUE
TRAINING MODE	PLYOMETRIC OR SPORT MIXED	10	6	FALSE	TRUE
TRAINING MODE	ECCENTRIC DOMINANT	8	6	FALSE	TRUE
TRAINING MODE	OTHER OR MIXED	2	2	FALSE	FALSE
STIFFNESS METHOD	COMMON ABSOLUTE RANGE	16	9	FALSE	TRUE
STIFFNESS METHOD	STUDY REPORTED OR UNCLEAR	21	11	FALSE	FALSE
STIFFNESS METHOD	INDIVIDUAL RELATIVE OR MAX	33	17	TRUE	TRUE

Moderator / contrast	Type	Estimate [95% CI]	df	p	FDR p
TENDON SITE; PATELLAR	CATEGORY GAP	−0.0686 [−0.1454, 0.0082]	19.63	0.077	—
TENDON SITE; ACHILLES	CATEGORY GAP	−0.0615 [−0.1316, 0.0085]	12.41	0.080	—
TENDON SITE; ACHILLES - PATELLAR	INTERACTION	0.0071 [−0.0951, 0.1092]	23.74	0.888	—
TENDON SITE; Omnibus	OMNIBUS	0.0071 [−0.0951, 0.1092]	23.74	0.888	0.888
TRAINING MODE; DYNAMIC RESISTANCE	CATEGORY GAP	−0.0572 [−0.1239, 0.0095]	17.38	0.088	—
TRAINING MODE; ECCENTRIC DOMINANT	CATEGORY GAP	−0.1238 [−0.3601, 0.1126]	4.38	0.227	—
TRAINING MODE; ISOMETRIC	CATEGORY GAP	−0.0200 [−0.1304, 0.0905]	5.86	0.673	—
TRAINING MODE; PLYOMETRIC OR SPORT MIXED	CATEGORY GAP	−0.0959 [−0.1859, −0.0060]	4.58	0.041	—
TRAINING MODE; ECCENTRIC DOMINANT - DYNAMIC RESISTANCE	INTERACTION	−0.0666 [−0.2646, 0.1314]	5.97	0.442	—
TRAINING MODE; ISOMETRIC - DYNAMIC RESISTANCE	INTERACTION	0.0372 [−0.0866, 0.1610]	9.22	0.515	—
TRAINING MODE; PLYOMETRIC OR SPORT MIXED - DYNAMIC RESISTANCE	INTERACTION	−0.0388 [−0.1492, 0.0717]	7.03	0.434	—

Moderator / contrast	Type	Estimate [95% CI]	df	p	FDR p
TRAINING MODE; Omnibus	OMNIBUS	F = 0.683	3, 10.26	0.582	0.873
STIFFNESS METHOD; INDIVIDUAL RELATIVE OR MAX	CATEGORY GAP	−0.0923 [−0.1820, −0.0026]	12.79	0.045	—
STIFFNESS METHOD; COMMON ABSOLUTE RANGE	CATEGORY GAP	−0.0036 [−0.1250, 0.1177]	7.29	0.946	—
STIFFNESS METHOD; COMMON ABSOLUTE RANGE - INDIVIDUAL RELATIVE OR MAX	INTERACTION	0.0886 [−0.0534, 0.2307]	14.09	0.202	—
STIFFNESS METHOD; Omnibus	OMNIBUS	0.0886 [−0.0534, 0.2307]	14.09	0.202	0.607

The training-mode omnibus test used the approximate Hotelling's T^2-Z (HTZ) small-sample Wald test (numerator df = 3; denominator df = 10.26). After source-confirmed label correction, the unchanged ≥8-unit/≥5-cohort rule retained dynamic resistance, eccentric-dominant, isometric and plyometric/sport-mixed categories; other/mixed remained descriptive. Respective participant-sample counts were 35, 8, 9 and 10; categories can share participants and cohorts, so these counts are not additive. The formal comparison comprised 68 matched units, 60 participant samples and 36 cohorts drawn from the unchanged 70-unit primary dataset. Single-contrast tests used cohort-clustered CR2 and Satterthwaite df. Category-gap p values test each gap against zero, not differences between training modes. FDR p values are Benjamini–Hochberg adjustments across the three formal moderator omnibus tests, not category-specific or pairwise adjustments. Low effective df and imprecise interactions limit interpretation; these findings provide limited evidence of moderation, not equivalence.

Targeted training-mode correction: source descriptions identified U19 and U20 as explosive and sustained isometric knee-extension training (Massey 2018, Methods pp. 3–4), and U32 as high-load cyclic isometric plantar-flexion training (Epro 2017, intervention section). U63 denotes endurance resistance training plus plyometrics (Kearns 2026, section 2.2 and Appendix A); its mixed-training category did not change. U64/U65 remained HRPT/HRT. Only three category assignments changed; no post hoc category merging was performed. Primary effect sizes and all other frozen analyses were unchanged. The audit and before/final category mappings accompany the reproducibility package.

Table S3 Available-data control-adjusted sensitivity

This reviewer-requested exploratory analysis includes 18 intervention units, 18 intervention samples, 11 cohorts and 11 unique concurrent control groups. It differs from controlled-design restriction, which retains within-group changes. The final primary control-adjusted delta is −0.131906 (95% CI −0.251101 to −0.012711; CR2 df 8.616; $p = 0.033817$). Its direction is supportive but its causal interpretation is limited by the small independent evidence base, low df, heterogeneous comparators and the interval crossing zero under $\rho = 0.90$.

Study	Comparator	Control n	Intervention units	Control source
Malliaras 2013	NON TRAINING CONTROL	9	3	Table 1 sample n; Table 2, p.1897, force and 75-100% stiffness
Massey 2018	NON TRAINING CONTROL	12	2	Publisher Table 1 footnote; Figures 6-7: ECT 13, SCT 15, CON patellar 12
Walker 2020	HABITUAL TRAINING CONTROL	8	2	Table 1, MVC and absolute stiffness; Subjects and Statistical Analyses sections
Eriksen 2019	USUAL ACTIVITY CONTROL	10	2	Table 2, CON n=10, maximal force and maximal stiffness; footnote means +/- SE
Hirayama 2017	USUAL ACTIVITY CONTROL	10	1	Table 1; Subjects n=10 control; 12-week endpoint
Jacobs 2025	ACTIVE ENDURANCE CONTROL	9	1	Table 1, control knee-extension moment and patellar stiffness; Participants/Study design
Laurent 2020	USUAL ACTIVITY CONTROL	10	2	Results p.1506; Subjects p.1504; Statistical Analyses means and SD
Bohm 2021	HABITUAL TRAINING CONTROL	10	1	Bohm 2021 Table 1; DOI 10.1098/rspb.2020.2784
Centner 2019	NON TRAINING CONTROL	13	2	Results pp.1663-1664; Table 1, completer n=13; mean +/- SD
Epro 2017	NON TRAINING CONTROL	13	1	Table 1, p.1013/PDF p.6; Statistics p.1011/PDF p.4
Reeves 2003	NON TRAINING CONTROL	9	1	Table 1, p.975/PDF p.5; Subjects and experimental design; stiffness 90-100% force

Scenario	r / rho	Delta [95% CI]	Cohorts	df	p
CONTROL ADJUSTED MAIN	0.7 / 0.5	−0.1319 [−0.2511, −0.0127]	11	8.62	0.034
WITHIN GROUP IDENTICAL SUBSET	0.7 / 0.5	−0.0910 [−0.2248, 0.0428]	11	8.72	0.158
RHO 0	0.7 / 0	−0.1332 [−0.2509, −0.0155]	11	8.52	0.031
RHO 0.3	0.7 / 0.3	−0.1329 [−0.2506, −0.0151]	11	8.58	0.031
RHO 0.7	0.7 / 0.7	−0.1304 [−0.2533, −0.0076]	11	8.60	0.040
RHO 0.9	0.7 / 0.9	−0.1282 [−0.2569, 0.0006]	11	8.51	0.051
R 0.3	0.3 / 0.5	−0.1276 [−0.2471, −0.0081]	11	7.54	0.039
R 0.5	0.5 / 0.5	−0.1314 [−0.2490, −0.0138]	11	8.10	0.033
R 0.9	0.9 / 0.5	−0.1284 [−0.2521, −0.0047]	11	8.76	0.044

Scenario	r / rho	Delta [95% CI]	Cohorts	df	p
SUPPLEMENT ONLY EXCLUDE HABITUAL ACTIVE COMPARATORS	0.7 / 0.5	−0.1662 [−0.3035, −0.0288]	8	6.04	0.025

Albracht 2013 meets the study-design definition of a controlled study but does not provide an eligible concurrent paired F+K control dataset in the frozen extraction; it therefore does not enter this available-data control-adjusted analysis. No control outcome was invented. Other controlled-design studies without usable paired control data were not imputed into this analysis. Comparator-specific provenance and group means/SDs are supplied in the control extraction CSV.

Table S4 Retained electronic search strategies

These entries combine retained database search records with complete contemporaneous instructions recovered for the three focused EBSCO searches. Platforms, final search dates, coverage, restrictions and retrieval totals are preserved. PubMed, Web of Science and SPORTDiscus retain their recorded strategies. For CINAHL Ultimate, Rehabilitation & Sports Medicine Source and Academic Search Premier, the TI and AB blocks and final combination below reproduce the original instructions dated 14 August 2026; they were not reconstructed from shorthand or memory. Database-tagged exports corroborate the totals. TI means title, AB abstract, SU subject and TS topic; N2 is the recorded proximity operator.

PubMed / MEDLINE

Field	Retained record
Platform	NCBI PubMed E-utilities
Final search date	2026-08-13
Coverage	Database inception to 2026-08-13
Filters / restrictions	Publication date through 2026-08-13; no language/species filter at search stage
Records retrieved	7586

(("Tendons"[Mesh] OR tendon*[Title/Abstract] OR "Achilles tendon"[Title/Abstract] OR "patellar tendon"[Title/Abstract] OR "quadriceps tendon"[Title/Abstract] OR "tendon stiffness"[Title/Abstract] OR "tendon mechanical properties"[Title/Abstract] OR "tendon strain"[Title/Abstract] OR "tendon modulus"[Title/Abstract] OR "tendon elasticity"[Title/Abstract]) AND ("Resistance Training"[Mesh] OR train*[Title/Abstract] OR "resistance training"[Title/Abstract] OR "strength training"[Title/Abstract] OR isometric*[Title/Abstract] OR eccentric*[Title/Abstract] OR concentric*[Title/Abstract] OR plyometric*[Title/Abstract] OR "jump training"[Title/Abstract] OR loading[Title/Abstract] OR "mechanical loading"[Title/Abstract] OR "cyclic strain"[Title/Abstract] OR exercise*[Title/Abstract]) AND (adapt*[Title/Abstract] OR longitudinal[Title/Abstract] OR "training-induced"[Title/Abstract] OR "pre post"[Title/Abstract] OR pretest[Title/Abstract] OR posttest[Title/Abstract] OR intervention*[Title/Abstract] OR "before and after"[Title/Abstract] OR effect*[Title/Abstract] OR change*[Title/Abstract] OR response*[Title/Abstract] OR plasticity[Title/Abstract] OR hypertroph*[Title/Abstract] OR "time course"[Title/Abstract])) AND ("1800/01/01"[Date - Publication] : "2026/08/13"[Date - Publication])

Web of Science

Field	Retained record
Platform	Institutional interface/API
Final search date	2026-08-14
Coverage	Database inception to 2026-08-14
Filters / restrictions	No language, species, or document-type filter; all years through formal search date
Records retrieved	13945

TS=(tendon* OR "Achilles tendon*" OR "patellar tendon*" OR "quadriceps tendon*" OR "tendon stiffness" OR "tendon mechanical properties" OR "tendon strain" OR "tendon modulus" OR "tendon elasticity") AND TS=(train* OR "resistance training" OR "strength training" OR isometric* OR eccentric* OR concentric* OR plyometric* OR "jump training" OR loading OR "mechanical loading" OR "cyclic strain" OR exercise*) AND TS=(adapt* OR longitudinal OR "training-induced" OR "pre post" OR pretest OR posttest OR intervention* OR "before and after" OR effect* OR change* OR response* OR plasticity OR hypertroph* OR "time course")

SPORTDiscus

Field	Retained record

Field	Retained record
Platform	EBSCOhost institutional interface
Final search date	2026-08-14
Coverage	Database inception to 2026-08-14
Filters / restrictions	No language filter; no full-text filter; no peer-reviewed filter; no date restriction
Records retrieved	3323

(TI (tendon* OR "Achilles tendon*" OR "patellar tendon*" OR "quadriceps tendon*" OR "tendon stiffness" OR "tendon mechanical properties" OR "tendon strain" OR "tendon modulus" OR "tendon elasticity") OR AB (tendon* OR "Achilles tendon*" OR "patellar tendon*" OR "quadriceps tendon*" OR "tendon stiffness" OR "tendon mechanical properties" OR "tendon strain" OR "tendon modulus" OR "tendon elasticity") OR SU (tendon* OR "Achilles tendon*" OR "patellar tendon*" OR "quadriceps tendon*" OR "tendon stiffness" OR "tendon mechanical properties" OR "tendon strain" OR "tendon modulus" OR "tendon elasticity")) AND (TI (train* OR "resistance training" OR "strength training" OR isometric* OR eccentric* OR concentric* OR plyometric* OR "jump training" OR loading OR "mechanical loading" OR "cyclic strain" OR exercise*) OR AB (train* OR "resistance training" OR "strength training" OR isometric* OR eccentric* OR concentric* OR plyometric* OR "jump training" OR loading OR "mechanical loading" OR "cyclic strain" OR exercise*) OR SU (train* OR "resistance training" OR "strength training" OR isometric* OR eccentric* OR concentric* OR plyometric* OR "jump training" OR loading OR "mechanical loading" OR "cyclic strain" OR exercise*)) AND (TI (adapt* OR longitudinal OR "training-induced" OR "pre post" OR pretest OR posttest OR intervention* OR "before and after" OR effect* OR change* OR response* OR plasticity OR hypertroph* OR "time course") OR AB (adapt* OR longitudinal OR "training-induced" OR "pre post" OR pretest OR posttest OR intervention* OR "before and after" OR effect* OR change* OR response* OR plasticity OR hypertroph* OR "time course") OR SU (adapt* OR longitudinal OR "training-induced" OR "pre post" OR pretest OR posttest OR intervention* OR "before and after" OR effect* OR change* OR response* OR plasticity OR hypertroph* OR "time course"))

CINAHL Ultimate

Field	Retained record
Platform	EBSCOhost institutional interface
Final search date	2026-08-14
Coverage	Database inception to 2026-08-14
Filters / restrictions	No language filter; no full-text filter; no peer-reviewed filter; no date restriction
Records retrieved	137

S1 TI (Achilles N2 tendon* OR patellar N2 tendon* OR quadriceps N2 tendon*) OR AB (Achilles N2 tendon* OR patellar N2 tendon* OR quadriceps N2 tendon*)

S2 TI (stiffness OR mechanical N2 propert* OR strain* OR modulus OR elastic*) OR AB (stiffness OR mechanical N2 propert* OR strain* OR modulus OR elastic*)

S3 TI (train* OR exercis* OR intervention* OR longitudinal OR resistance N2 train* OR strength N2 train* OR isometric* OR eccentric* OR concentric* OR plyometric* OR jump N2 train* OR mechanical N2 load*) OR AB (train* OR exercis* OR intervention* OR longitudinal OR resistance N2 train* OR strength N2 train* OR isometric* OR eccentric* OR concentric* OR plyometric* OR jump N2 train* OR mechanical N2 load*)

Final combination S1 AND S2 AND S3

Source: contemporaneous project instructions, 14 August 2026, 11:58:06 UTC. Supplied settings: Boolean/Phrase; no language, publication-date, full-text, peer-reviewed or human filter; strength was not mandatory. The recorded 137 CINAHL Ultimate records agree with database attribution in the retained import log.

Rehabilitation & Sports Medicine Source

Field	Retained record
Platform	EBSCOhost institutional interface
Final search date	2026-08-14
Coverage	Database inception to 2026-08-14
Filters / restrictions	No language filter; no full-text filter; no peer-reviewed filter; no date restriction
Records retrieved	184

S1 TI (Achilles N2 tendon* OR patellar N2 tendon* OR quadriceps N2 tendon*) OR AB (Achilles N2 tendon* OR patellar N2 tendon* OR quadriceps N2 tendon*)

S2 TI (stiffness OR mechanical N2 propert* OR strain* OR modulus OR elastic*) OR AB (stiffness OR mechanical N2 propert* OR strain* OR modulus OR elastic*)

S3 TI (train* OR exercis* OR intervention* OR longitudinal OR resistance N2 train* OR strength N2 train* OR isometric* OR eccentric* OR concentric* OR plyometric* OR jump N2 train* OR mechanical N2 load*) OR AB (train* OR exercis* OR intervention* OR longitudinal OR resistance N2 train* OR strength N2 train* OR isometric* OR eccentric* OR concentric* OR plyometric* OR jump N2 train* OR mechanical N2 load*)

Final combination S1 AND S2 AND S3

Source: contemporaneous project instructions, 14 August 2026, 11:58:06 UTC. Supplied settings: Boolean/Phrase; no language, publication-date, full-text, peer-reviewed or human filter; strength was not mandatory. The recorded 184 Rehabilitation & Sports Medicine Source records agree with database attribution in the retained import log.

Academic Search Premier

Field	Retained record
Platform	EBSCOhost institutional interface
Final search date	2026-08-14
Coverage	Database inception to 2026-08-14
Filters / restrictions	No language filter; no full-text filter; no peer-reviewed filter; no date restriction
Records retrieved	524

S1 TI (Achilles N2 tendon* OR patellar N2 tendon* OR quadriceps N2 tendon*) OR AB (Achilles N2 tendon* OR patellar N2 tendon* OR quadriceps N2 tendon*)

S2 TI (stiffness OR mechanical N2 propert* OR strain* OR modulus OR elastic*) OR AB (stiffness OR mechanical N2 propert* OR strain* OR modulus OR elastic*)

S3 TI (train* OR exercis* OR intervention* OR longitudinal OR resistance N2 train* OR strength N2 train* OR isometric* OR eccentric* OR concentric* OR plyometric* OR jump N2 train* OR mechanical N2 load*) OR AB (train* OR exercis* OR intervention* OR longitudinal OR resistance N2 train* OR strength N2 train* OR isometric* OR eccentric* OR concentric* OR plyometric* OR jump N2 train* OR mechanical N2 load*)

Final combination S1 AND S2 AND S3

Source: contemporaneous project instructions, 14 August 2026, 11:58:06 UTC. Supplied settings: Boolean/Phrase; no language, publication-date, full-text, peer-reviewed or human filter; strength was not mandatory. The recorded 524 Academic Search Premier records agree with database attribution in the retained import log.

Citation chasing and source accounting

Scopus was not searched because access was unavailable. The final WoS forward export contained 410 records, comprising 375 exact master matches and 35 genuinely new records; all 35 were excluded at title/abstract screening. The backward pass traversed 2,219 cited-reference links across 55 matched seeds, representing 1,274 unique references. A 60-reference key-review check identified 58 exact matches, one already-included title variant and Quinlan 2021. A supplemental Quinlan pass traversed 75 PubMed-indexed backward and 23 forward records with no additional eligible cohort. Seed coverage was not complete for all intended WoS seeds. The earlier Europe PMC graph pass was not repeated.

Final effective flow	PubMed	Additional databases + retained citations
Records entering effective pathway	7,586	9,276 + 8
Duplicates removed	2	1 late duplicate
Records screened	7,584	9,283
Title/abstract exclusions	7,408	9,276
Reports sought	176	7
Reports not retrieved	11	1
Reports assessed	165	6
Reports excluded after report assessment	114	0
Included reports	51	6

The six databases contributed 25,699 raw records. The five additional databases contributed 18,113 raw records, 8,837 exact existing-master matches and 9,276 new records. The additional operational queue combined 602 database-retained and seven citation-retained records (609 total): 602 title/abstract-decisive exclusions, five included reports, one not retrieved and one duplicate. Subsequent citation chasing added one included report. The figure reassigns the duplicate before effective screening and never treats the operational queue as 609 full texts assessed. Eight retained citation records are not the total citation records identified.

Table S5 Frozen outcome-selection hierarchy

Rule	Frozen selection
Achilles strength	Maximum Achilles tendon force derived from the matched MVC test; then plantar-flexion MVC/isometric peak torque used for mechanics testing; then another anatomically matched maximal endpoint only if neither is available.
Patellar strength	Maximum patellar/quadriceps tendon force derived from the matched MVC test; then knee-extension MVC/isometric peak torque; then another anatomically matched maximal endpoint only if higher-priority measures are unavailable.
Stiffness	Authors' prespecified eligible target-tendon force–elongation slope under active loading; if common absolute and timepoint-relative ranges are both available, prefer the common absolute high-force range; otherwise the report's primary high-force linear-region estimate. Young's modulus is not substituted.
Bilateral / within-person	Retain frozen arm effects with the same participant-sample identity; limbs do not create independent participant samples. No averaging or duplicated participant n solely to remove dependence.
Multiple endpoints / ranges	One primary strength and one primary stiffness endpoint per biological arm, tendon site and timepoint. Alternate eligible endpoints/ranges remain non-primary and are not averaged into a new independent effect.
Timepoint	First formal post-training endpoint after the training period; longer follow-up is non-primary. Epro POST_78W does not enter the first-post primary set.
Strict maximal strain	Maximal voluntary contraction, maximal tendon force, maximal loading or 100% maximal torque. Common-force strain alone is not automatically maximal strain. Same-trial linkage to the chosen strength endpoint requires source documentation and was not uniformly recorded.
Companion reports	Fill missing information on the same biological key; never create a second independent effect. Resolve duplicate numerical reports using corrected supplement/table, primary table, results text, then reliable figure recovery; do not average conflicting reports.
Source-level application	Vikmoen's anatomically unrelated composite 1RM endpoint was removed under the matching rule. Albracht 2013 was treated independently from Bohm 2021, with maximal Achilles force preferred to joint torque. These approved corrections were completed before final analysis freeze.

Strict-strain provenance

Study	Tendon	Retained definition	Source location
Reeves 2003	Patellar	Strain at maximal tendon force	Full-text table
Werkhausen 2018	Achilles	Strain at maximal common force	Results text
Eriksen 2019	Patellar	At maximum patellar tendon force	Table 2
Eriksen 2019	Patellar	At maximum patellar tendon force	Table 2
Arampatzis 2007	Achilles	Maximum Achilles tendon strain during MVC/maximal tendon force.	Table 1
Arampatzis 2007	Achilles	Maximum Achilles tendon strain during MVC/maximal tendon force.	Table 1
Epro 2017	Achilles	Maximal Achilles tendon strain during maximal loading.	Table 1
Lee 2023	Patellar	Peak patellar tendon elongation divided by resting length during 100% RMVC/maximal tendon force.	Table 5

Study	Tendon	Retained definition	Source location
Lee 2023	Patellar	Peak patellar tendon elongation divided by resting length during 100% RMVC/maximal tendon force.	Table 5
Geremia 2018	Achilles	Maximal elongation divided by resting tendon length at maximal ramp MVIC force.	PDF p.1732, Table 2
Carroll 2011	Patellar	Patellar tendon strain at peak/maximal tendon force.	PMC3154687 Table 5
Carroll 2011	Patellar	Patellar tendon strain at peak/maximal tendon force.	PMC3154687 Table 5
Carroll 2011	Patellar	Patellar tendon strain at peak/maximal tendon force.	PMC3154687 Table 5
Bontemps 2024	Patellar	Maximum elongation divided by resting tendon length at ramp MVC	Table 3
Lambrianides 2024	Achilles	Maximum elongation divided by resting tendon length at 100% maximal tendon force	Table 1
Lambrianides 2024	Achilles	Maximum elongation divided by resting tendon length at 100% maximal tendon force	Table 1
Nulty 2025	Patellar	Maximum elongation divided by resting tendon length at ramp MVC	Table 3
Nulty 2025	Patellar	Maximum elongation divided by resting tendon length at ramp MVC	Table 3
Lee 2025	Patellar	Maximum strain during ramp MVC/maximal tendon force	Table 3
Lee 2025	Patellar	Maximum strain during ramp MVC/maximal tendon force	Table 3
Malliaras 2013	Patellar	Strain at maximum tendon force during maximal knee-extension loading	Table 2
Malliaras 2013	Patellar	Strain at maximum tendon force during maximal knee-extension loading	Table 2
Malliaras 2013	Patellar	Strain at maximum tendon force during maximal knee-extension loading	Table 2
Albracht 2013	Achilles	Maximum GM tendon-aponeurosis strain during maximal voluntary isometric plantar flexion	Table 1/Statistics p.1609; Subjects and mechanics pp.1607-1608

The row count is 24 effects, not 24 independent cohorts. The frozen primary subset contains 22 participant samples and 14 cohorts. The records preserve source definitions without newly inferring that all endpoints used the exact same contraction trial. Detailed biological keys remain in strict_strain_data.csv.

Supplementary Statistical Methods

Longitudinal effects and variance

$$lnRR = ln(meanpost / meanpre)$$

$$v = \frac{SDpre^2}{n \times meanpre^2} + \frac{SDpost^2}{n \times meanpost^2} - \frac{2r \times SDpre \times SDpost}{n \times meanpre \times meanpost}$$

$$r = (SDpre^2 + SDpost^2 - SDchange^2) / (2 \times SDpre \times SDpost)$$

$$SD = SEM \times \sqrt{n}$$

Reported pre/post correlation takes priority, followed by valid derivation from change-score dispersion. Otherwise r = 0.70 is used at effect-size calculation, with r = 0.30, 0.50 and 0.90 sensitivities. No r was imputed during extraction. The primary effect requires both valid lnRR means and its sampling variance. Missing post SD is not equated to change SD or supplied through an equal-SD rule.

Matched multilevel model

$$y(c,s,u,o) = \mu(o) + b(c) + a(c,s) + h(c,s,u,o) + e(c,s,u,o)$$

Outcome indicators without an intercept estimate strength and stiffness means. The model contains a cohort random intercept, participant-sample random intercept and outcome-specific DIAG heterogeneity within matched unit. The primary contrast is $\delta = \mu strength - \mu stiffness$. Within-unit sampling covariance is $\rho\sqrt{(vstrength \times vstiffness)}$; $\rho = 0.50$ is primary and 0, 0.30, 0.50, 0.70 and 0.90 form the frozen sensitivity grid. Unknown sampling covariances between different units are zero in the working matrix; hierarchical random effects and cohort-clustered CR2 address dependence without identifying those unreported covariances. Fitting uses REML and inference uses cohort-level CR2 with Satterthwaite df.

Control-adjusted transformation

$$Ladjusted,o = Ltraining,o - Lcontrol,o$$

$$V = B\,\Sigma\,B^{\mathrm{T}}$$

Σ contains the variances and working cross-outcome covariance of each unique training/control estimate. Each row of B has +1 for the training estimate and −1 for its control. The same control appears once in Σ even when multiple contrasts use it, so $B\Sigma B^{\mathrm{T}}$ generates the induced shared-control covariance. Control n is not split or multiplied. The control-adjusted paired gap is the adjusted strength lnRR minus adjusted stiffness lnRR. Comparator conditions may include habitual activity and are not uniformly inactive.

Heterogeneity and prediction interval

Component	Variance	Heterogeneity SD
cohort	0.0041014825	0.064042817
participant sample	1.580471e-11	3.9755138e-06
strength DIAG	0.0020320863	0.045078668
stiffness DIAG	0.020273269	0.14238423

$$PI = \delta \pm t(0.975, \nu) \times \sqrt{[SECR2(\delta)^2 + \tau strength^2 + \tau stiffness^2]}$$

The reporting-only model-conditional interval is −0.375 to 0.243 for the true contrast in a new matched unit under the frozen hierarchy. Shared cohort/sample intercepts cancel in the matched difference; outcome-specific variances add

under DIAG. This approximation does not include uncertainty in heterogeneity estimates and is not a unique clinical prediction interval across arbitrary populations or training programs. No model was refitted for this derivation.

Small-study effects and moderator inference

$$g = ystrength - ystiffness$$

$$vg = vstrength + vstiffness - 2\rho\sqrt{}(vstrength \times vstiffness)$$

The frozen diagnostic regresses g on its working standard error √vg using the dependence-aware multilevel framework and cohort-CR2 inference, with 37 independent cohorts. The slope is −1.3907 (95% CI −4.3380 to 1.5566; df 14.75; p = 0.330). It provides no clear evidence of small-study effects, but limited power, heterogeneity and working covariance assumptions prevent concluding that publication bias is absent. Moderator tests retain frozen thresholds/reference categories; the multi-parameter HTZ test is the approximate Hotelling's T^2-Z small-sample Wald test. Non-significant findings are not equivalence tests.

Internal specifications and subsequent changes

The review and statistical analyses were completed without prior public protocol registration, and the review remains unregistered. The retained calculation and meta-model contracts document internal staged specifications, but no authenticated prospective protocol date is claimed. Original files are included in the reproducibility package; later filesystem timestamps are not evidence of prospective planning. The inventory below describes known changes without inventing prospective dates.

Item	Relationship to primary specification
Internal calculation/model contracts	Retained staged specifications; not a prospective registry record.
Active-adjunct sensitivity	Additional sensitivity identified separately from the primary within-group estimand.
Reviewer-requested control adjustment	Added for revision using available concurrent controls; exploratory, not replacement of the primary estimand.
Endpoint and cohort source corrections	Removed an ineligible composite endpoint; separated Albracht/Bohm; included independent Albracht force/stiffness and strict strain before final freeze.
Prediction interval and natural-scale CIs	Reporting derivations from frozen estimates/variance components; no change to the fitted model.

Figure S1

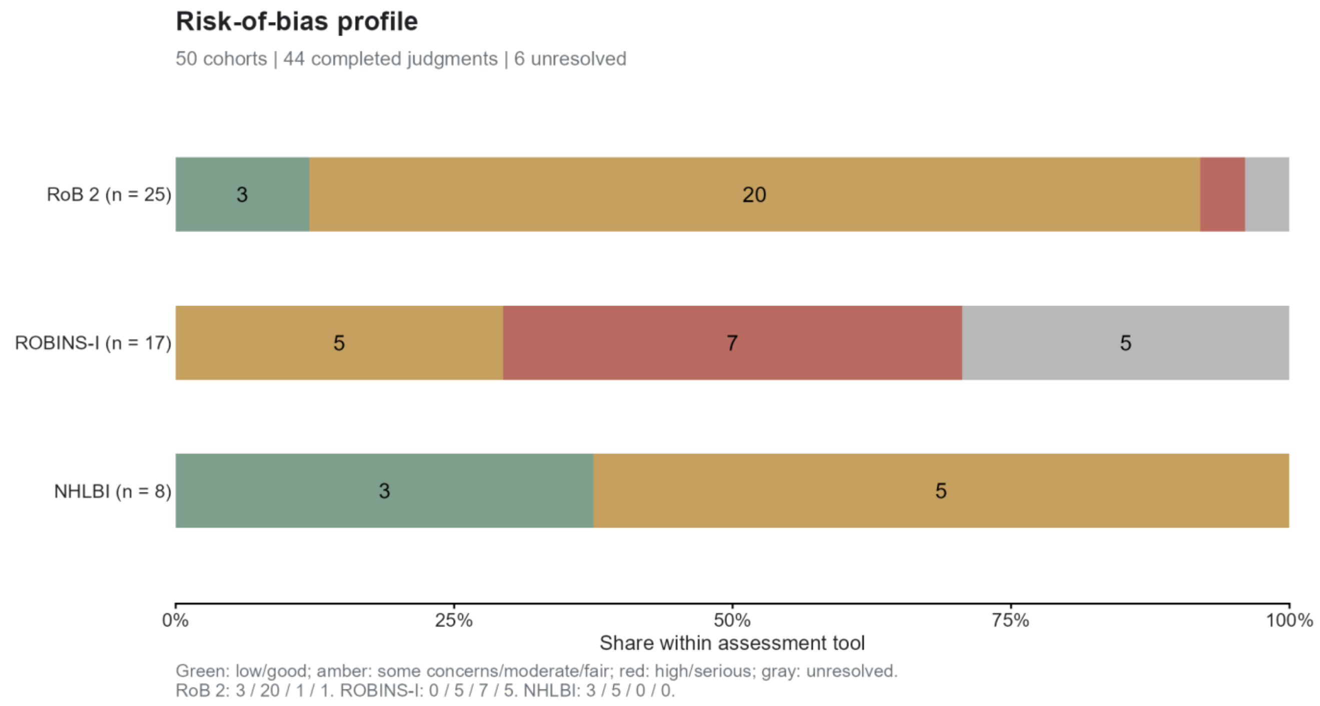

Figure S1. Corrected tool-specific RoB profile across 50 cohorts. Forty-four judgments are complete and six are unresolved. Bohm 2021 and Albracht 2013 are separate, author-confirmed assessments. No cross-tool numeric quality score is used.

Figure S2

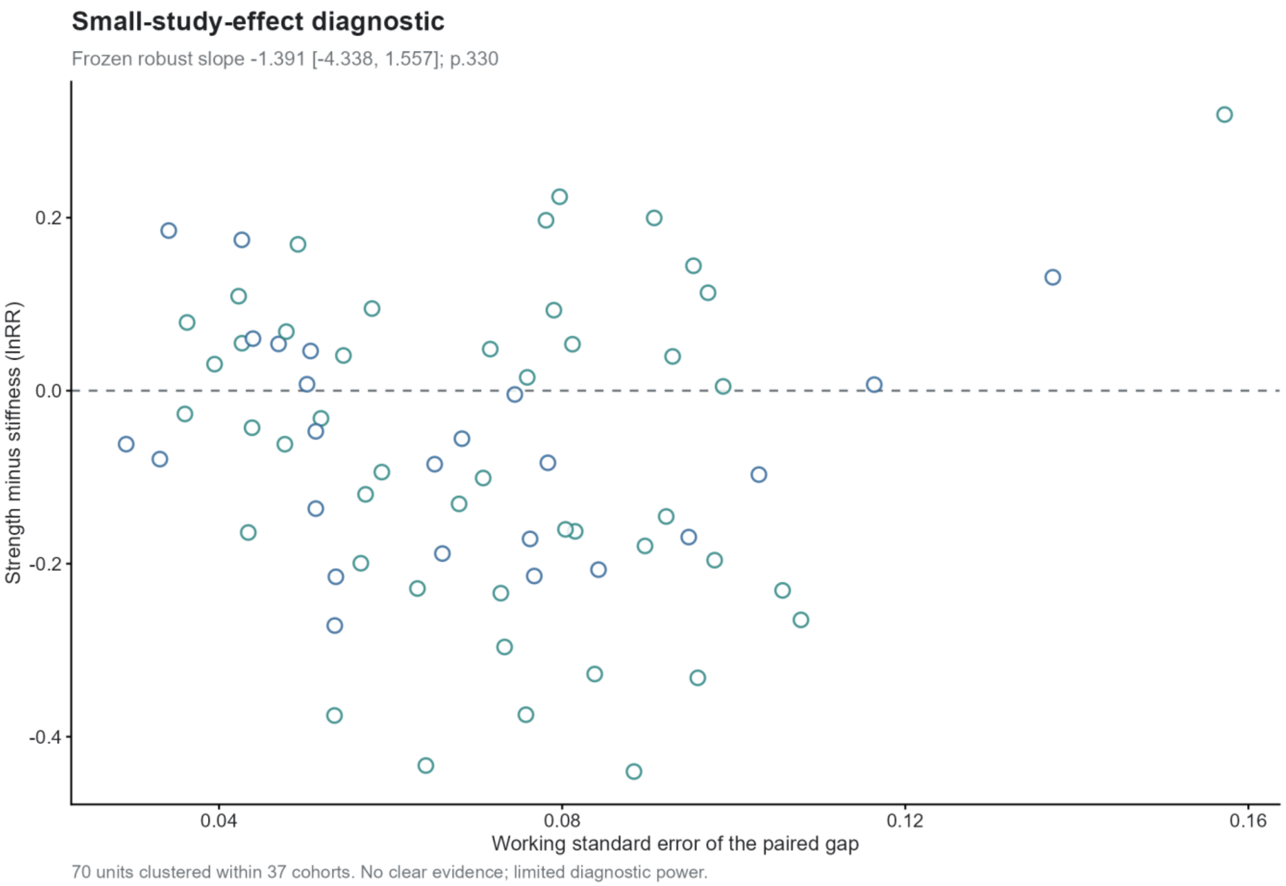

Figure S2. Dependence-aware small-study diagnostic with 70 units clustered within 37 cohorts. The frozen robust slope is reported on the figure; no ordinary independent-study Egger test was fitted. The graphic is descriptive and does not establish publication bias or its absence.

Figure S3

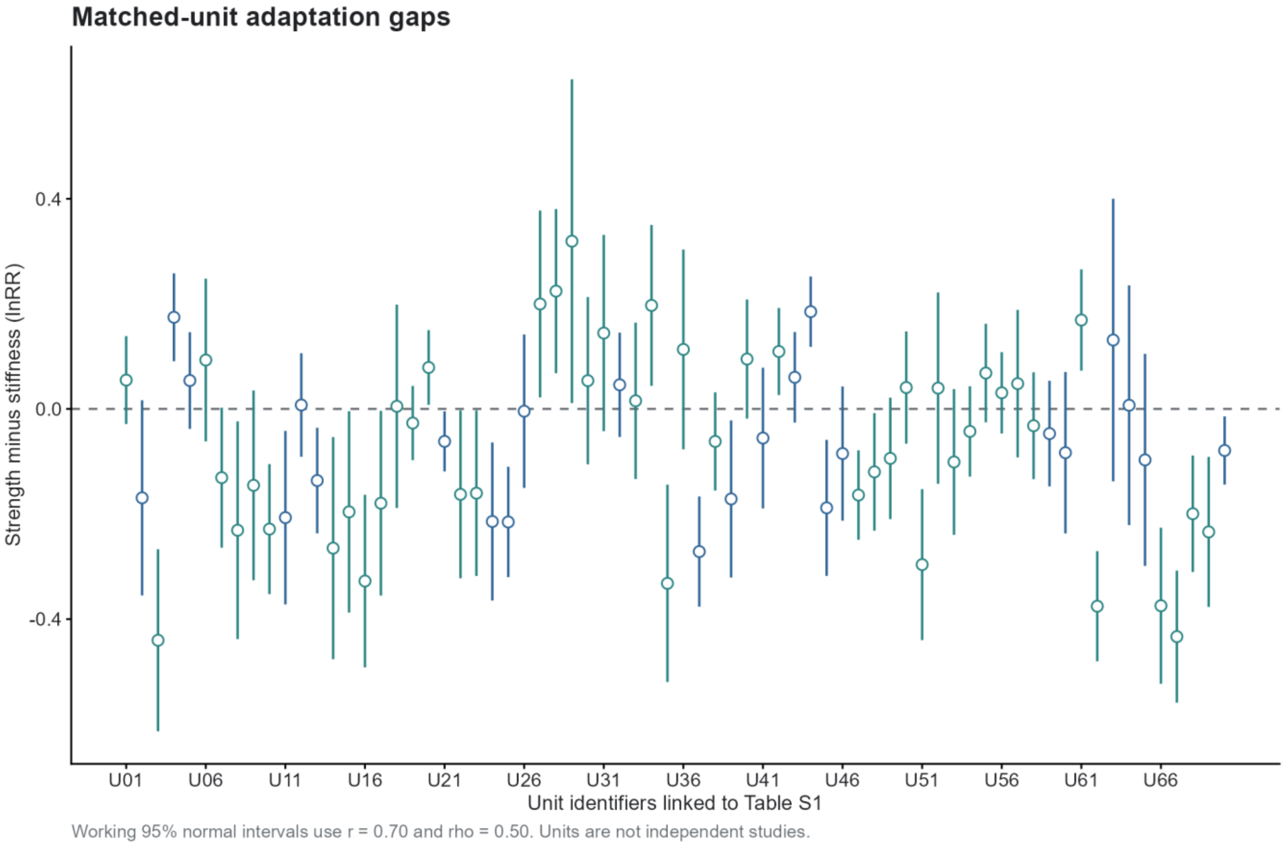

Figure S3. Matched-unit gaps with working normal 95% intervals using the frozen r = 0.70 and ρ = 0.50 variances. U identifiers map to Table S1 and the biological-key CSV. Units sharing participants/cohorts are not independent studies.

Table S6 Selected apparently eligible reports excluded from the review

These examples are reproduced from the final screening decisions, without new eligibility assessment. Each publication is identified by its title and PubMed identifier. Numbers in square brackets refer to the main manuscript reference list. The list is illustrative rather than a second flow count; included reports that lacked usable numerical data are not reclassified as excluded.

Report title	Identifier	Recorded exclusion reason
Effects of minute oscillation stretching training on muscle and tendon stiffness and walking capability in people with type 2 diabetes.	PMID 39249539 [93]	Population did not meet healthy/asymptomatic eligibility.
Addressing muscle-tendon imbalances in adult male athletes with personalized exercise prescription based on tendon strain.	PMID 38842575 [94]	Population did not meet healthy/asymptomatic eligibility.
Effects of a 12-week gait retraining program on the Achilles tendon adaptation of habitually shod runners.	PMID 37817483 [95]	No eligible anatomically matched strength outcome.
Quantification of patellar tendon strain and opportunities for personalized tendon loading during back squats.	PMID 37248376 [96]	No eligible longitudinal training intervention.
In Achilles Tendinopathy the Symptomatic Tendon Differs from the Asymptomatic Tendon While Exercise Therapy Has Little Effect on Asymmetries-An Ancillary Analysis of Data from a Controlled Clinical Trial.	PMID 36769750 [97]	Population did not meet healthy/asymptomatic eligibility.
Metabolic and molecular responses of human patellar tendon to concentric- and eccentric-type exercise in youth and older age.	PMID 35948859 [98]	No eligible target-tendon stiffness outcome.
Effects of Eccentric Single-Leg Decline Squat Exercise on the Morphological and Structural Properties of the Vastus Lateralis and Patellar Tendon.	PMID 33333987 [99]	No eligible target-tendon stiffness outcome.

Table S7 Cohort risk of bias and synthesis profiles

Judgments below reproduce risk_of_bias_FINAL.csv. RoB 2, ROBINS-I and NHLBI labels retain their tool-specific meaning and are not a common quality scale or certainty-of-evidence grade. The public ledger supplies domain judgments, supporting evidence and source locations. No formal outcome-level certainty assessment was conducted.

Synthesis	Cohorts	Tool-specific profile
Primary F+K	37	NHLBI before-after / fair: 5; NHLBI before-after / good: 3; ROBINS_I_2016 / moderate: 1; ROBINS_I_2016 / serious: 6; RoB 2 / high: 1; RoB 2 / low: 3; RoB 2 / some concerns: 18
Control-adjusted	11	NHLBI before-after / fair: 1; NHLBI before-after / good: 1; ROBINS_I_2016 / serious: 1; RoB 2 / low: 1; RoB 2 / some concerns: 7
Strict strain	14	NHLBI before-after / fair: 3; NHLBI before-after / good: 2; ROBINS_I_2016 / serious: 1; RoB 2 / low: 2; RoB 2 / some concerns: 6

The synthesis profiles refer to the distinct cohorts contributing to each indicated frozen dataset. The same cohort can contribute to more than one synthesis. Covariance scenarios and leave-one-cohort-out refits do not constitute newly assessed studies. Study characteristics and formal moderator category sizes are given in Tables S1–S3.

Study / cohort	Design	Tool / overall judgment	Contribution
Seynnes 2009 C006_Seynnes2009_patellar	uncontrolled single group prepost	NHLBI before-after / fair	Primary F+K
Reeves 2003 C009_REEVES2003	uncontrolled single group prepost	NHLBI before-after / fair	Primary F+K; Control-adjusted; Strict strain
Kubo 2012 C013_KUBO2012	uncontrolled single group prepost	NHLBI before-after / good	Primary F+K
Geremia 2018 C017_GEREMIA2018	uncontrolled single group prepost	NHLBI before-after / good	Primary F+K; Strict strain
Werkhausen 2018; Werkhausen 2019 C019_Werkhausen2018_Achilles	uncontrolled single group prepost	NHLBI before-after / fair	Primary F+K; Strict strain
Epro G 2017 C1B_EPRO2017	uncontrolled single group prepost	NHLBI before-after / good	Primary F+K; Control-adjusted; Strict strain
Bontemps B 2024 C1B1_BONTEMPS2024	uncontrolled single group prepost	NHLBI before-after / fair	Primary F+K; Strict strain
Onambele-Pearson GL 2012 C1B1_ONAMBELE2012	uncontrolled single group prepost	NHLBI before-after / fair	Primary F+K
Arampatzis 2007 C010_ARAMPATZIS2007	randomized within person	RoB 2 / some concerns	Primary F+K; Strict strain
Kongsgaard 2007 C011_KONGSGAARD2007	randomized within person	RoB 2 / some concerns	Primary F+K
Kubo 2017 C013_Kubo2017_Achilles	randomized within person	RoB 2 / some concerns	Primary F+K
Lambrianides Y 2024 C1B_LAMBRIANIDES2024	randomized within person	RoB 2 / some concerns	Primary F+K; Strict strain

Study / cohort	Design	Tool / overall judgment	Contribution
McMahon 2013 C011_McMahon2013_patellar	randomized parallel controlled	RoB 2 / some concerns	Primary F+K
Malliaras 2013 C014_MALLIARAS2013	randomized parallel controlled	RoB 2 / some concerns	Primary F+K; Control-adjusted; Strict strain
Kubo 2017 C016_KUBO2017_PT	randomized within person	RoB 2 / some concerns	Primary F+K
Massey 2018 C016_Massey2018_patellar	randomized parallel controlled	RoB 2 / some concerns	Primary F+K; Control-adjusted
Centner 2019 C018_CENTNER2019	randomized parallel controlled	RoB 2 / low	Primary F+K; Control-adjusted
Walker 2020 C022_Walker2020_patellar	randomized parallel controlled	RoB 2 / some concerns	Primary F+K; Control-adjusted
Carroll CC 2011 C1B_CARROLL2011	randomized parallel controlled	RoB 2 / low	Primary F+K; Strict strain
Eriksen CS 2018 C1B_ERIKSEN2018	randomized parallel controlled	RoB 2 / some concerns	Not in these syntheses
Eriksen CS 2019 C1B_ERIKSEN2019	randomized parallel controlled	RoB 2 / some concerns	Primary F+K; Control-adjusted; Strict strain
Fouré A 2013 C1B_FOURE2013	randomized parallel controlled	RoB 2 / some concerns	Not in these syntheses
Grosset JF 2014 C1B_GROSSET2014	randomized parallel controlled	RoB 2 / some concerns	Primary F+K
Hirayama K 2017 C1B_HIRAYAMA2017	randomized parallel controlled	RoB 2 / some concerns	Primary F+K; Control-adjusted
Jerger S 2023; Jerger S 2022 C1B_JERGER2022_2023	randomized parallel controlled	RoB 2 / high	Primary F+K
Laurent C 2020 C1B_LAURENT2020	randomized parallel controlled	RoB 2 / some concerns	Primary F+K; Control-adjusted
Lee J 2023 C1B_LEE2023_U21	randomized parallel controlled	RoB 2 / some concerns	Primary F+K; Strict strain
Lee J 2025 C1B_LEE2024_PRO	randomized parallel controlled	RoB 2 / some concerns	Primary F+K; Strict strain
Nulty CD 2025; Nulty 2024 C1B_NULTY2025	randomized parallel controlled	RoB 2 / low	Primary F+K; Strict strain
Balshaw TG 2023 C1B1_BALSHAW2023	randomized parallel controlled	RoB 2 / some concerns	Primary F+K
Quinlan 2021 C1C2_QUINLAN2021	randomized parallel controlled	RoB 2 / some concerns	Primary F+K
Ben Jeddou 2018 C1C1_BENJEDDOU2018	randomized parallel controlled	RoB 2 / unresolved source insufficient	Not in these syntheses
McMahon 2018 C017_McMahon2018_patellar	nonrandomized parallel controlled	ROBINS_I_2016 / serious	Primary F+K
Jacobs MW 2025 C1B_JACOBS2025	nonrandomized parallel controlled	ROBINS_I_2016 / serious	Primary F+K; Control-adjusted

Study / cohort	Design	Tool / overall judgment	Contribution
Vesterhus I 2025 C1B_VESTERHUS2025	nonrandomized parallel controlled	ROBINS_I_2016 / serious	Primary F+K
Domroes T 2024 C1B1_DOMROES2024	nonrandomized parallel controlled	ROBINS_I_2016 / serious	Primary F+K
Kearns 2026; Kearns 2022 C1C1_KEARNS2026	nonrandomized parallel controlled	ROBINS_I_2016 / serious	Primary F+K
Vikmoen O 2016 C1B_VIKMOEN2016	nonrandomized parallel controlled	ROBINS_I_2016 / serious	Not in these syntheses
Kay AD 2024 C1B1_KAY2024	nonrandomized parallel controlled	ROBINS_I_2016 / moderate	Not in these syntheses
Waugh 2018; Waugh 2021 C019_WAUGH2018	nonrandomized parallel controlled	ROBINS_I_2016 / unresolved source insufficient	Not in these syntheses
Cesanelli L 2026 C1B_CESANELLI2026	nonrandomized parallel controlled	ROBINS_I_2016 / unresolved source insufficient	Not in these syntheses
Fouré A 2010 C1B_FOURE2010	nonrandomized parallel controlled	ROBINS_I_2016 / unresolved source insufficient	Not in these syntheses
Létocart AJ 2024 C1B_LETOCART2024	nonrandomized parallel controlled	ROBINS_I_2016 / unresolved source insufficient	Not in these syntheses
Arampatzis 2010 C012_ARAMPATZIS2010	nonrandomized within person	ROBINS_I_2016 / moderate	Not in these syntheses
Bohm 2014 C015_BOHM2014	nonrandomized within person	ROBINS_I_2016 / moderate	Not in these syntheses
Kubo K 2002 C1B1_KUBO2002	nonrandomized within person	ROBINS_I_2016 / moderate	Primary F+K
Tsai MS 2024 C1B1_TSAI2024	nonrandomized within person	ROBINS_I_2016 / moderate	Not in these syntheses
Moltubakk 2021 C1C1_MOLTUBAKK2021	nonrandomized within person	ROBINS_I_2016 / unresolved source insufficient	Not in these syntheses
Bohm S 2021 C1B_BOHM2021	randomized parallel controlled	RoB 2 / some concerns	Primary F+K; Control-adjusted
Albracht 2013 C_ALBRACHT2013	nonrandomized parallel controlled randomization not reported	ROBINS_I_2016 / serious	Primary F+K; Strict strain
